# Silk sericin and its biomedical applications: extraction methods, genetic insights (Ser1–Ser6), and developments of multifunctional biomaterials for tissue regeneration

**DOI:** 10.3389/fbioe.2026.1835198

**Published:** 2026-09-03

**Authors:** P. Thamaraiselvi, Sunita Nayak

**Affiliations:** School of Bioscience and Technology, Vellore Institute of Technology, Vellore, Tamil Nadu, India

**Keywords:** biocompatible, biomaterials, glycoprotein, scaffolds, sericin, tissue engineering

## Abstract

Sericin protein is derived from silk cocoons from silkworms. Sericin functions as a glue to bond the two fibroin filaments together in the silk cocoon. It is a hydrophilic, hot water-soluble macromolecular glycoprotein. In the silk industry, sericin is usually removed and discarded as waste during the degumming process of silk yarn or fabrics, often by boiling with soap water. Besides being readily available and inexpensive, sericin is biocompatible and biodegradable. In recent times, sericin has received considerable attention due to its numerous versatile properties and potential applications in various areas, including cosmetics, pharmaceuticals, food, sustained drug delivery, and the fabrication of functional biomaterials, particularly in the biomedical field. Sericin, as a biomaterial with biocompatibility, immune-compatibility, biodegradability, anti-inflammatory, antibacterial, antioxidant, and photoprotective properties, has been identified as a potential biomaterial. This review aims to outline and contextualize recent advancements in sericin research, with a special focus on tissue engineering applications. These include insight into sericin gene expression in the silkworm (Ser1 to Ser6) and the use of sericin as a biomaterial in various forms, such as films, fibers, sponges, gels, and bio-coatings for implants, as well as micro/nano vehicles for targeted delivery systems. Although sericin is mainly described as a waste-derived biomaterial, this review attempts to integrate sericin gene diversity, extraction strategies, and structure–function relationships to identify the key determinants of its translational potential for tissue engineering applications.

## Graphical Abstract

**Figure:**
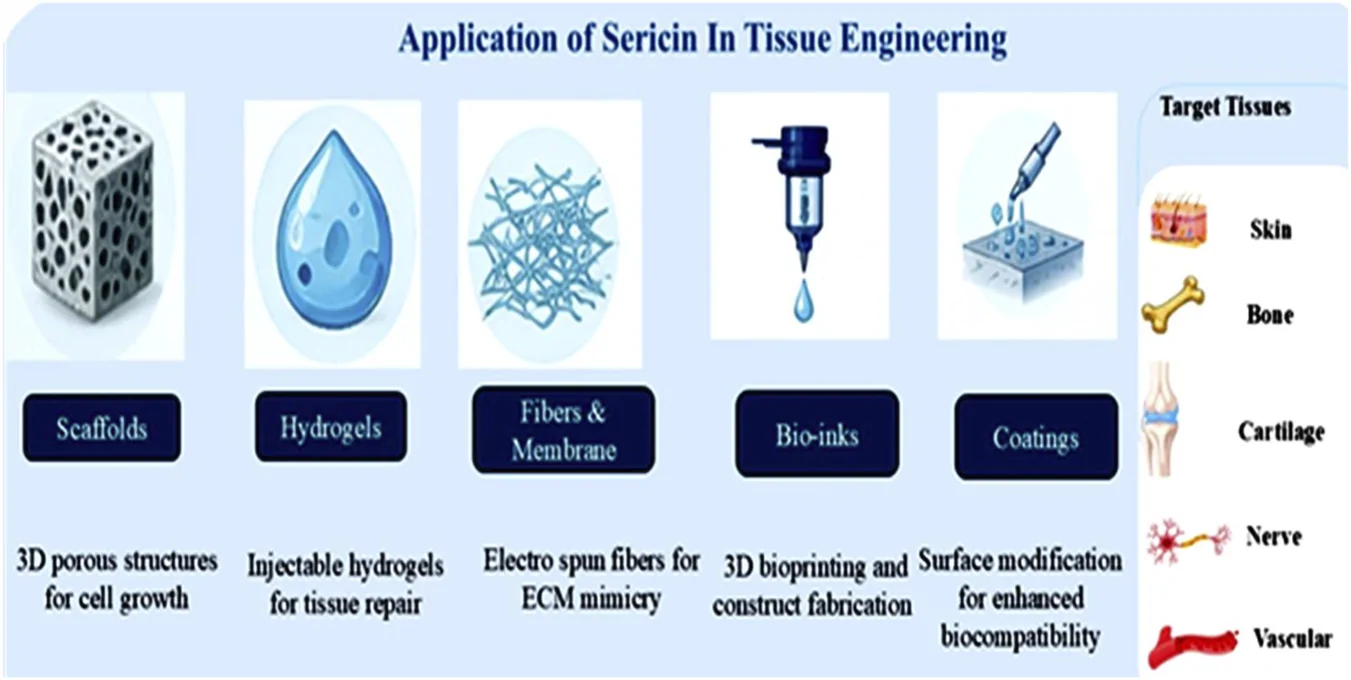

## Highlights

- Sericin, a natural biocompatible protein, promotes cell attachment and proliferation, making it ideal for biomedical applications.
- The *Bombyx mori* genome contains six genes (Ser1 to Ser6), each of which plays a crucial role in the protein’s structural and functional properties.
- Sericin has been developed in various bulk and micro/nano forms, enhancing its tissue engineering potentials to repair or replace damaged tissue

## 1 Introduction

Electronic databases, like as PubMed, Scopus, Web of Science, and Google Scholar, were used to conduct a literature search for identifying relevant studies related to sericin. The articles published up to 2026 were focused primarily on highlighting recent advances in sericin. Moreover, landmark studies published, were included to provide the necessary fundamental background information. Recently, sericin has demonstrated significant potential as an effective biopolymer with inherent properties that make it suitable for various biomedical applications. Sericin is obtained through different extraction methods from the silk industry waste. The scale-up of silk sericin (SS) recovery from textile waste is cost-effective due to the abundance of sericin, which constitutes + approximately 20%–30% of silk mass and results in an annual waste of around 50,000 tons/year worldwide. Analyzing the process reveals that recovering sericin from degumming wastewater can lead to competitive unit costs compared to commercial sericin, with potential reductions of about 30% through process optimization. However, energy and labor costs remain significant factors (Wang et al., 2021; Capar and Pilevneli, 2024).

**FIGURE 1:**
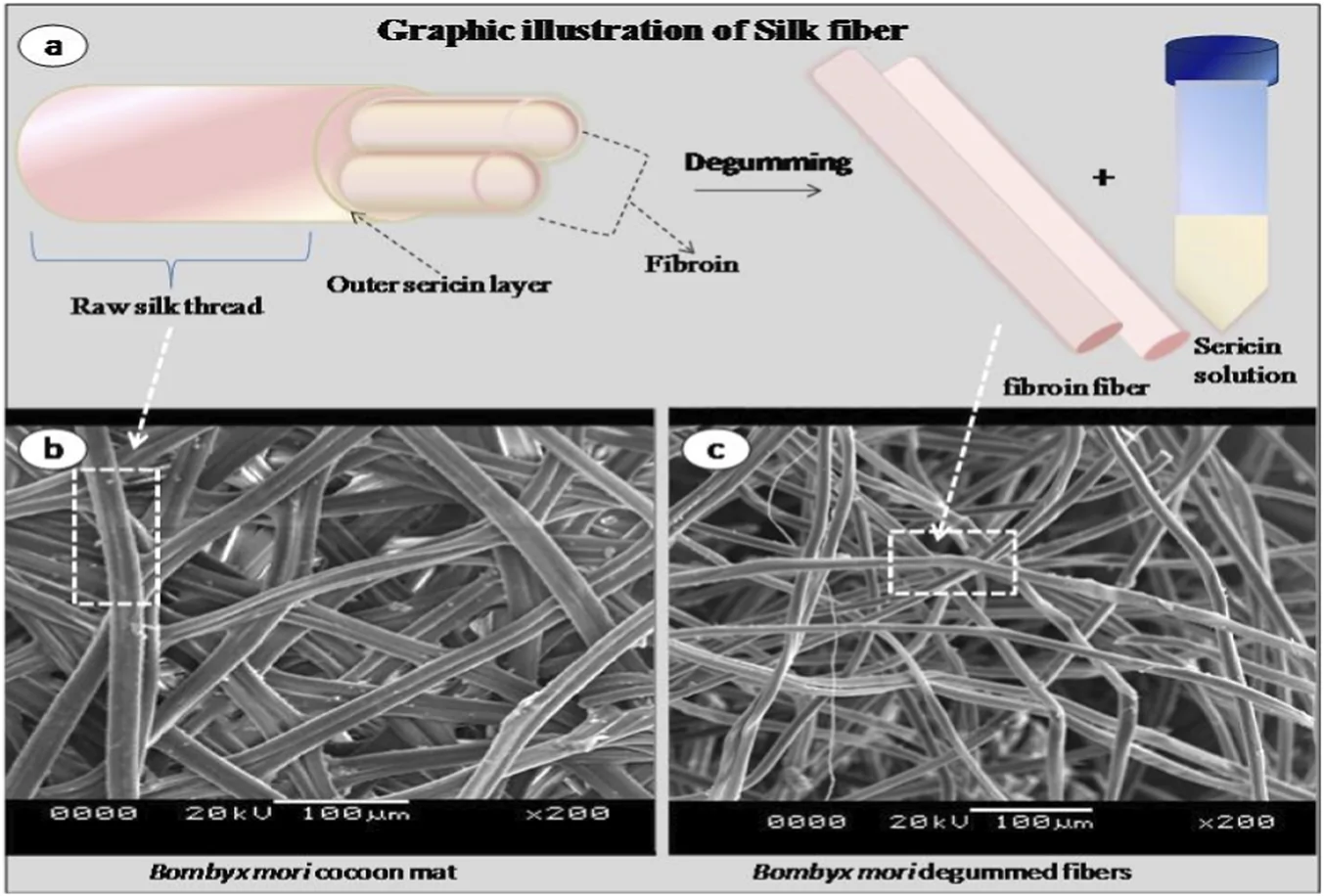
Graphic illustration of silk filament; **(a)** raw silk thread undergoing the process of degumming, **(b)** SEM image of *Bombyx mori* silk fibers in cut cocoon mat, **(c)** *Bombyx mori* degummed silk fibers without sericin.

Sericin has been found to possess antioxidant, antibacterial, anticancer, and anti-tyrosinase properties, offering promising applications in food, pharmaceuticals, cosmetics, and, more recently, in regenerative medicine and tissue engineering. Sericin is derived from unprocessed silk cocoon fibers of silkworms belonging to the species Lepidoptera larvae. Silkworms of the families Bombycidae and Saturniidae produce marketable silks. Silks are commonly classified into non-mulberry and mulberry types. Mulberry silkworms are mainly of the Bombycidae family (e.g., *Bombyx mori*), domesticated and specially fed on mulberry trees. Conversely, non-mulberry silkworms belong to the Saturniidae family (e.g., *Antheraea mylitta, Antheraea assamensis, Antheraea polyphemus, Antheraea yamamai, Antheraea proylei, Antheraea pernyi*) and feed on non-mulberry leaves, either wild or semi-domesticated. Natural silk fibres from silkworms consist of two primary proteins: ‘sericin’ and ‘fibroin’. Two fibroin filaments form the hydrophobic core, surrounded by a layer of the hydrophilic glue protein “sericin,” as shown in Figure 1. Sericin biosynthesis occurs in the middle silk glands (MSG) of silkworms, with molecular weights ranging from 10 to over 400 kDa (Kunz et al., 2016).

Sericin constitutes approximately 10%–30% of the total protein weight in a cocoon from various silkworm species. However, sericin can be extracted in large quantities up to 98.5% from a mutant variety of *B. mori* known as ‘Sericin Hope’. It consists of 18 different amino acids, notably the polar side groups like carboxyl and hydroxyl, with aspartic acid making up about 16.7% and serine around 33.4%, which contribute to its high hydrophilicity (Zhang et al., 2012). The glue-like quality of sericin is due to extensive intermolecular hydrogen bonds formed between hydroxyl groups of its serine residues and the structural fibroin proteins. Additionally, the presence of multiple functional groups in sericin, such as hydroxyl, carboxyl, and amino groups, allows for manipulation (copolymerization, mixing, and cross-linking) with other polymers to create new materials for specific applications across various fields (Kim et al., 2023). Enriched with glycine, serine, and aspartic acid, amorphous silk sericin (SS) is highly hydrophilic and forms a water-soluble random coil structure; however, it turns into a slightly soluble or insoluble state while transitioning into a dense, crystalline β-sheet structure during drying, processing, or at lower temperatures. Its properties make it potentially valuable for cosmetic, pharmaceutical, biotechnological, and medical uses, as it is widely recognized for UV resistance, moisture retention, biocompatibility, antibacterial effects, anti-tyrosinase activity, anticoagulant, and anti-cancer properties (Aad et al., 2024). This review discusses the detailed characteristics of silk sericin as a biomaterial for tissue engineering and regenerative medicine, especially in skin regeneration. It emphasizes sericin’s potential in promoting skin wound healing through its antibacterial, antioxidant, and anti-inflammatory properties, which are essential for effective tissue repair. The development of sericin-based biomaterials is often simple and environmentally sustainable, suited for widespread application in wound dressings.

## 2 Mulberry and non-mulberry silkworms

### 2.1 Mulberry silk sericin

Sericin proteins produced by *B. mori*, a mulberry silkworm, have a molecular weight ranging from 20 to 400 kDa. Particularly, it has a high percentage of serine (40%) and a substantial amount of glycine (16%) (Lamboni et al., 2015). *Bombyx mori* sericin exhibits a broad, heterogeneous molecular-weight distribution, typically spanning from low-kDa peptides to several hundred kDa, with the exact HPLC/SEC profile depending strongly on the extraction method and degree of hydrolysis (Yang et al., 2014). *B*. *mori* silk fiber exhibits primarily 400, 250, and 150 kDa sericin proteins, the adhesive protein that coats the core fibroin filaments. The molecular weight of sericin can significantly influence its biological properties and industrial applications (Seo et al., 2023; Aad et al., 2024). Higher molecular weight characteristically improves mechanical strength for scaffolds, and lower molecular weight favors pharmaceutical or cosmetic uses. From the cocoon of *B. mori*, in 1980, Tokutake isolated the smallest component of 24 kDa sericin fragment dissolved in an ethylenediamine and cupric hydroxide solution. The middle silk gland (MSG) of *B. mori* contains five types of sericin polypeptides, s-1, s-2, s-3, s-4, and s-5, with molecular weights ranging from 80 to 310 kDa. These sericin components are central in the development of the cocoon layers during the final stage of larval development. From the middle section of MSG, s-1 and s-3 are synthesized, while s-2 and s-5 are produced from the anterior section, and s-4 is produced from the posterior section of the MSG (Tokutake, 1980). *Bombyx mandarina,* which is the wild ancestor of *B. mori* and contributes genetic variability through interbreeding (Yang et al., 2014).

### 2.2 Non-mulberry silk sericin

The peduncle of tropical tasar silkworm *Antheraea mylitta* has yielded sericin with a molecular weight of 200 kDa, primarily composed of amino acids such as serine, glutamic acid, glycine, threonine, and tyrosine. Dash et al. (2006) reported three main proteins from the cocoon of *A. mylitta*, weighing 70 kDa, 200 kDa, and over 200 kDa (Dash et al., 2007; Dash et al., 2006). Additionally, the sericin proteins extracted from wild-type varieties (*A. mylitta* and *A. assamensis*) were found to differ biochemically from *B. mori*. Similarly, from cocoons, sericin proteins of 400 kDa from *Cricula trifenestrata*, 41 kDa, from *A. yamamai*, and 66 kDa from *A. assamensis* and *Philosamia ricini* have been isolated (Cui et al., 2009). There are differences in degumming ratios between non-mulberry species (6%–11%) and mulberry (18%–21%) when using the non-chemical autoclave method. Sericin yields from non-mulberry species vary significantly and are highly unpredictable, as the different sericin yields depend on the sericin content in mulberry and non-mulberry silk cocoons (Kalita et al., 2022). Table 1 shows the different molecular weights of silk sericin protein isolated from different silkworms.

**TABLE 1:** Molecular weight of silk sericin protein isolated from different silkworms.

Silkworm type	Source	Molecular weight (kDa)	Ref.
Mulberry			
*B. mori*	Gland	80–310	Prudhomme et al. (1985), Michaille et al. (1989)
*Bombyx mori*	Cocoon	10–400	Takasu et al. (2002), Silva et al. (2012)
Sericin Hope	Cocoon	400, 250, 150	Mase et al. (2006)
Non-Mulberry			
*Samia cynthia* *Antheraea pernyi*	Cocoon Middle silk gland	7.08 ≈35 kDa and ≈240 kDa	Endrawati et al. (2023) Wei et al. (2025)
*Gonometa postica and* *Gonometa rufobrunnea*	Cocoon	10–400,25–1,150	Kanyora et al. (2024)
*A. mylitta*	Cocoon	70, 200, >200	Dash et al. (2007)
	Peduncle	200	Dash et al. (2006)
	Gland	30, >50, 70, 200, >200	Kundu et al. (2008)
*A. assama*	Cocoon	66	Ahmad et al. (2004)
*P. ricini*	Cocoon	66	Ahmad et al. (2004)
*C. trifenestrata*	Cocoon	400	Yamada et al. (2001)
*A. yamamai* *Hyalophora cecropia* *A. atlas*	Cocoon Cocoon Cocoon	41 10–400 kDa 8.99	Cui et al. (2009) Aad et al. (2024) Sherova et al. (2023)

## 3 Gene and protein organization of sericin

**FIGURE 2:**
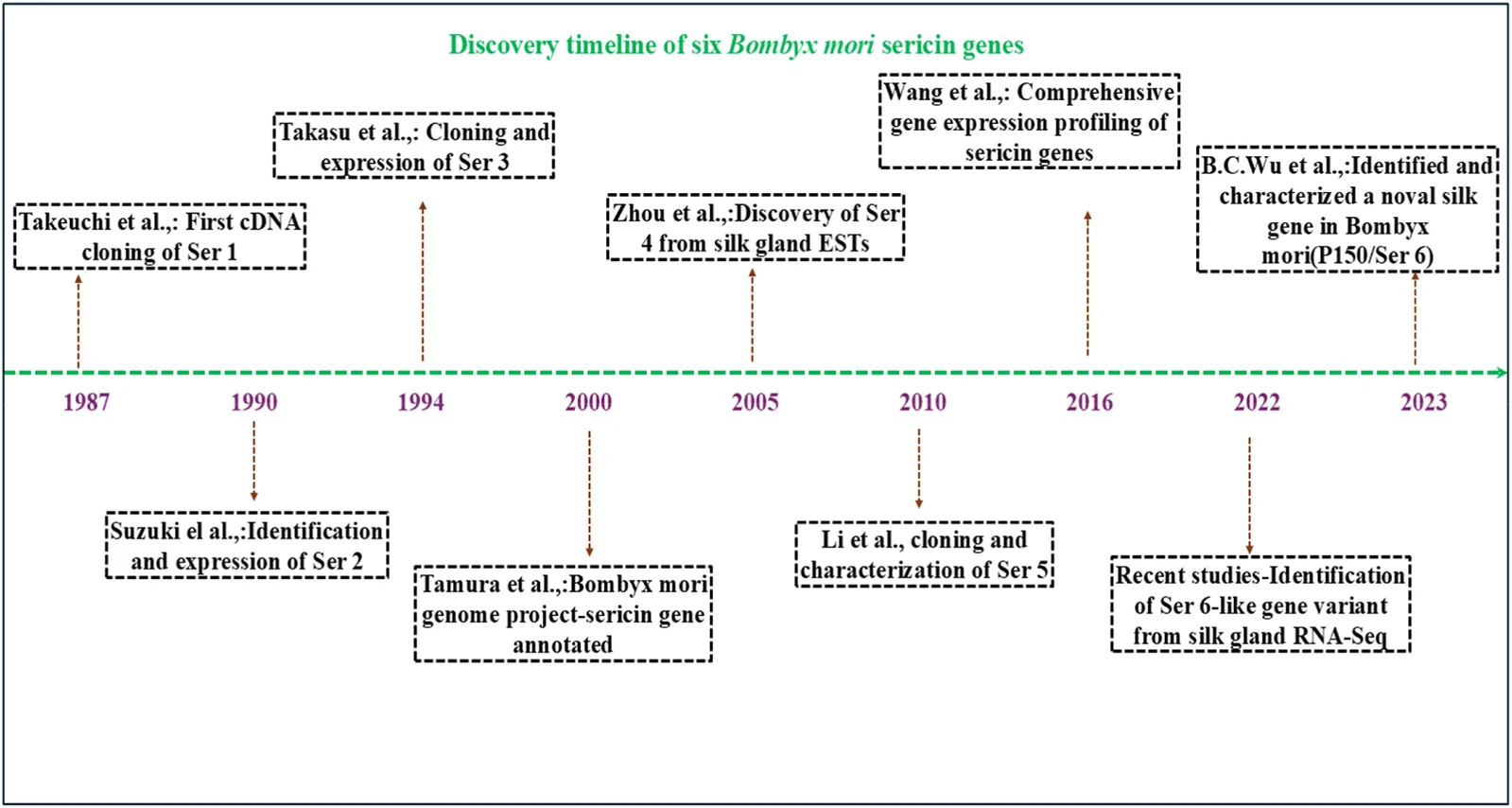
Structured timeline chart summarizing the discovery and key features of six known sericin genes in *Bombyx mori*.

The sericin gene comprises of multiple exons and introns, which allow the production of wide-ranged sericin proteins through alternative splicing that encode both crystalline (ordered) and non-crystalline (amorphous or random coil) protein regions. Among 6 and 15 estimated sericin-type proteins isolated from *B. mori* silk, at the molecular level, five sericin genes, Ser1, Ser2, Ser3, Ser4 and Ser5 have been identified. Sericin 1 (Ser1) and Sericin 3 (Ser3) are the primary components responsible for binding silk fibers within the cocoon, whereas Sericin 2 (Ser2), Sericin 4 (Ser4), and Sericin 5 (Ser5) are associated with non-cocoon silk produced by younger larvae (Garel et al., 1997). The exons of the Ser gene encode parts of crystalline proteins and regions of non-crystalline proteins, and selective mRNA splicing produces proteins with different structural domains. This splicing process can generate several protein variants of exons encoding Ser mRNA at various stages of larval development (Couble et al., 1987). Subsequently, in the same individual, each transcription can produce proteins with diverse structures and features. The molecular characterization of sericin has developed gradually and six sericin genes have been identified in Bombyx mori. The relative chronology of their discovery and their major structural and functional features are shown in Figure 2.

**FIGURE 3:**
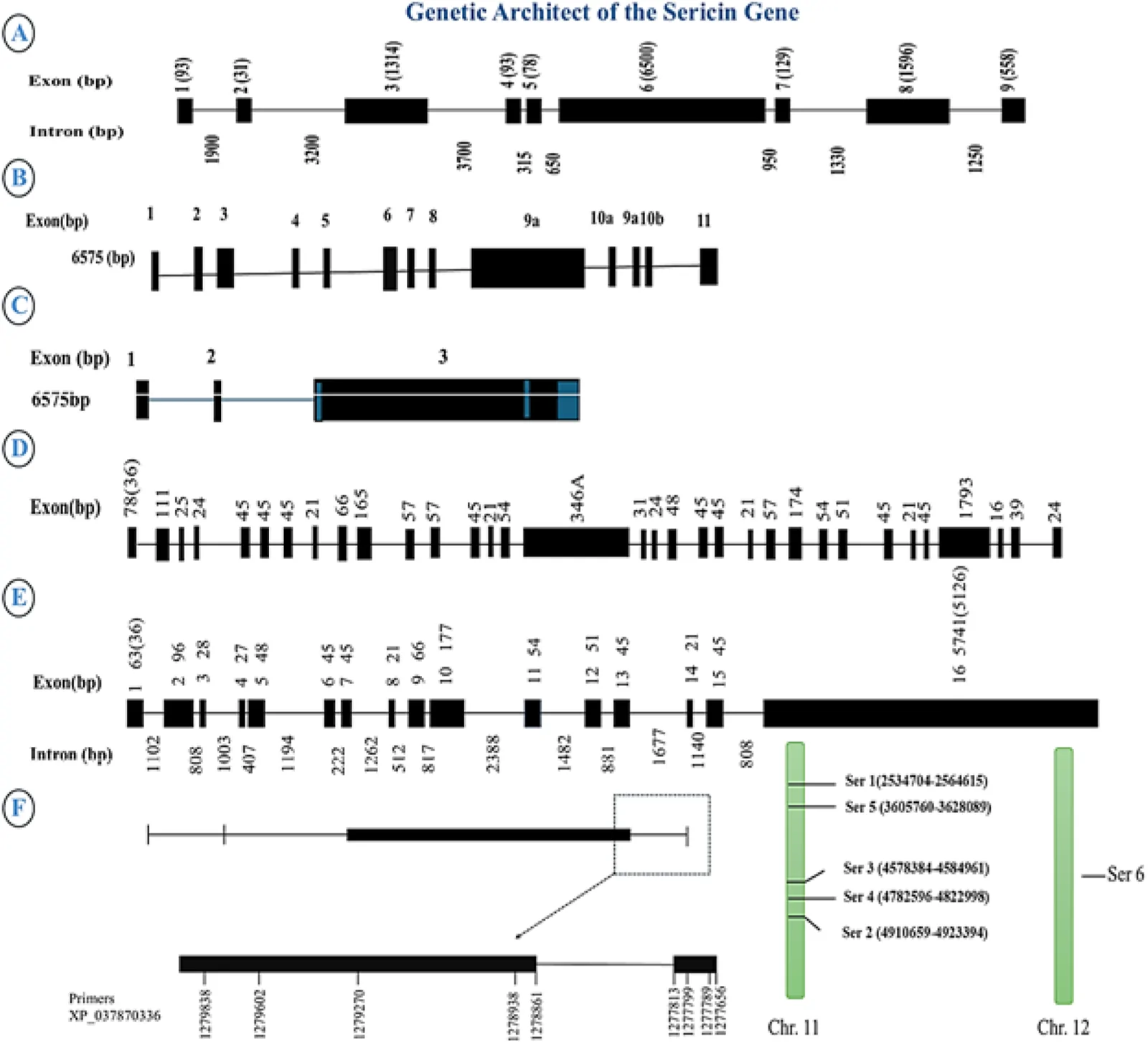
Genetic architecture of the silkworm sericin gene. **(A)** SER 1. **(B)** SER 2. **(C)** SER 3. **(D)** SER 4. **(E)** SER 5. **(F)** SER 6.

Ser1 in the silkworm *Bombyx mori* is located at the Src locus on chromosome 11, consists of 9 exons and 8 introns, and is approximately 23 kb long. The central alternative exon (exon 6) encodes 60 repeats of a unique 114-bp motif. It also contains two additional repeating exons (Garel et al., 1997; Shao et al., 2012). Through alternative splicing, it leads to the production of different combinations of exons, mainly four mRNA transcripts (10.5, 9.0, 4.0, and 2.8 kb) in different sections of the MSG, during the 5th larval instar (Kludkiewicz et al., 2009; Takasu et al., 2007). A tissue- and development-regulated mechanism causes alternative splicing of the Ser1 primary transcript (Garel et al., 1997; Couble et al., 1987). A set of constitutive exons (1, 2, 3, 5, 6, and 9) is present in all these major transcripts. Constitutive Exons of a total of 569 bp long, having 369 bp of coding sequence (123 codons) followed by 188 bp of non-coding sequence along with 7 repeats of 129 bp and 8 repeats of 114 bp, are present in all sericins (Figure 3A) (Michaille et al., 1986). The proteins corresponding to the four Ser1 mRNAs have molecular weights of 330, 284, 123, and 76 kDa. Exons 6 and 8 encode 40% of sericin-rich motifs, which form most of the β-sheet structure. Peptides with physicochemical and structural characteristics that facilitate sericin’s sliding and adhesion to fibroin are encoded by exons 3, 6, and 8, with peptide 3 having high water solubility (Kwak et al., 2017). Ser1 proteins in the MSG stored in the lumen as a “gel-like” solution exists in an amorphous random coil structure with a lower proportion of β-sheet structures. The Ser1 gene in *B. mori* encodes a protein with a repetitive 38-amino acid motif rich in hydrophilic amino acids like serine, threonine, and tyrosine. The high serine content (44.7%), presence of threonine (10.5%) and tyrosine (7.9%) enhance the protein’s hydrophilicity and interactions with other molecules. The high prevalence of hydroxyl-containing amino acids (serine, threonine) enables extensive hydrogen bonding and makes sericin highly hydrophilic, leading to the protein’s hydrophilic and “gluelike” properties. Overall, the secondary structure of natural sericin, is characterized by a random coil structure with some β-sheet regions, in the MSG or cocoon (Holm and Buschard, 2019).

Depending on the allele considered, the Daizo p50 strain of *B. mori* carries the Ser2 D allele, which was fully sequenced and submitted to the major databases the Ser 2 gene encodes either 6.4 or 5 and 3.1 kb mRNAs. Ser2 comprises 13 exons, ranging in size from 28 to 2,574 bp, indeed considered more complex and variable than the Ser1 gene due to its genomic structure and transcript diversity. The exon arrangement of the Ser 2 gene D allele generally follows the pattern 1, 2, 3, 4, 5, 6, 7, 8, 9a, 10a, 9b, 10b, and 11 (Figure 3B). The two mRNA variants produced by the Ser2 gene result from alternative splicing of exon 9a and are exclusively expressed in MSG (Kludkiewicz et al., 2009). The protein encoded by the longer Ser 2 splice variant has a molecular weight of 230 kDa, while the shorter form weighs 120 kDa. Silk fibres stickiness is highly attributed by Ser2 proteins due to abundance of charged amino acids, facilitating electrostatic interactions that hold the cocoon threads together. Only two of the fifteen amino acids within the repetitive motif of Ser2 proteins are serine residues (Guo et al., 2022). The charged residues in the Ser2 motif, including arginine, glutamic acid, aspartic acid, and lysine, contribute to the formation of random coil structures or less ordered, more soluble protein region suggesting that the repeated region likely does not form a β-sheet structure. Conclusively, Ser2 proteins serve different functions, such as attaching to surrounding materials, and do not form a rigid structure (Kludkiewicz et al., 2009) (Guo et al., 2022).

Ser3 found on chromosome 11, forms a non-spliced mRNA transcript of 4.5 kb reflecting the mRNA corresponding to sericin A. Compared to the Ser1 gene, which is highly complex with multiple splice variants, Ser3 is expressed as a single, simple transcript in the anterior part of the middle silk gland (Takasu et al., 2007). The Ser3 protein contains 1,271 amino acid residues, and it was hypothesised that the 18 residues at the N-terminus function as a signal peptide (Yamada et al., 2001; Chen et al., 2022). Ser 3 protein comprises unique repetitive motifs, of an 86-amino acid motif and an 8-amino acid repeat, both of which are comprised of high serine concentrations (>45% serine residues). The Ser3 protein’s hydrophobicity suggests it is more hydrophilic than the Ser1 protein, which implies that the Ser3 protein may have more fluidity since it retains more water than the Ser1 protein (Holm and Buschard, 2019; Wang Y. et al., 2026).

The recently identified sericin 4 gene is about 40 kb, containing 34 exons and six alternatively spliced transcripts (Figure 3C) (Guo et al., 2022). Silkworms produce two types of silk: cocoon silk with inner sericin layers of Ser1 and Ser3, and non-cocoon silk with outer sericin layers of Ser2 and Ser4 (and more recently identified Ser5) with proteins found in the larval silk spun before cocooning or when fixing the cocoon to a substrate (Figure 3D). Sericin 4 (Ser4) from *B. mori* shows strong adhesive properties due to its repetitive motifs rich in charged amino acids and glutamine, which enable it to form robust interactions with fibroin filaments and help maintain silk integrity during larval growth. Structurally, Ser4 is a high molecular weight protein (∼260–280 kDa) with domains abundant in serine, aspartic acid, glycine, and glutamine, facilitating extensive hydrogen bonds and electrostatic interactions critical for adhesion. These characteristics are linked to the increased stickiness seen in non-cocoon silks produced during the second to fourth larval instars. Functionally, unlike cocoon-specific sericins, Ser4 is vital in early development, acting as a biological adhesive that aids in the cohesive layering around fibroin fibers. This role is supported by its co-localization with Ser1 in the middle silk gland (MSG), shown through immunofluorescence. Due to its hydrophilic and polar nature, like other sericins (20–400 kDa), Ser4 has significant potential in biomaterials, especially in tissue engineering, where its adhesive properties and reported non-cytotoxicity can be utilized, along with its contribution to mechanical interlocking and strain resistance in silk-based structures (Dong et al., 2019). Recent research using PacBio Iso-Seq technology identified new transcripts and extended non-coding RNAs, improving understanding of silkworm transcriptomes and uncovering potential genes and regulatory proteins. Combining transcriptome and genome analyses led to the identification of the Sericin5 (Ser5) gene (Figure 3E). Its full sequence, confirmed to be on chromosome 11, exceeds 22 kb and includes 16 exons. RACE (Rapid Amplification of cDNA Ends) and transcriptome sequencing techniques helped to obtain its full-length cDNA, overcoming challenges posed by many repeat sequences. The study also examined Ser5’s transcriptional and translational expression patterns, shown in Figure 3E. Moreover, *B. mori’s* high-resolution pan-genome, which includes 7,308 newly discovered gene families, offers a comprehensive resource for exploring genetic diversity and understanding functions of recent genes, such as P150/sericin 6 (Figure 3F). The biocompatibility of sericin, including P150/Ser6, is well recognised, along with its potential biomedical. In *B. mori*, the P150/sericin 6 gene (P150/Ser6) is on chromosome 12; however, the specific chromosomal coordinates are not provided. Previous models for P150/Ser6 were inaccurate because they split the homologous sequence into two separate genes (from *G. mellonella* and *Escherichia kuehniella*), with unknown functions. The new model shows P150/Ser6 has four exons and is expressed in the middle silk gland. It is a large gene, about 20 kb, with four exons and three introns. The first two exons encode a signal peptide and part of a nonrepetitive sequence. The third exon is particularly large, containing two core repetitive sections flanked by different sequences and accounting for 94% of the open reading frame (ORF). The last exon encodes a short ORF and a stop codon. Its deduced protein is enormous, with 4,552 amino acids rich in polar amino acids primarily serine (Ser), glycine (Gly), and aspartic acid (Asp) along with tyrosine (Tyr) and a molecular weight of 467 kDa, characterised by a high number of repetitive structures. The *B. mori* Ser6 gene encodes a 4,552-amino acid protein (P150/SP150) containing 45 copies of a 30-amino acid repeat (Wu et al., 2024) and 73 copies of a 35-amino acid repeat (Wu et al., 2023).

## 4 Sericin extraction

**FIGURE 4:**
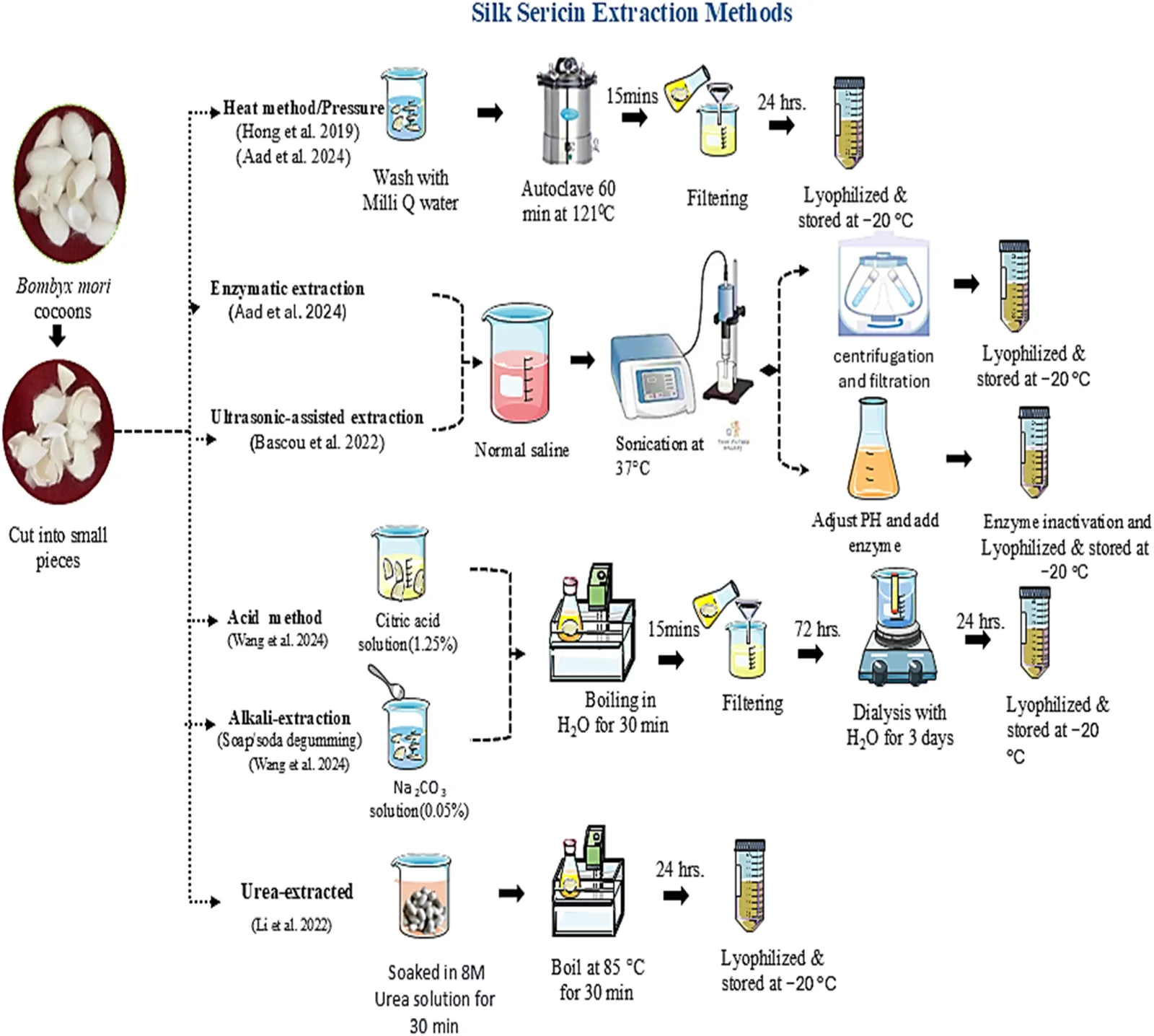
Different types of sericin extraction methods.

The use of sericin in recent years for various biomedical applications has grown significantly. Therefore, to use it, the sericin protein must be extracted from raw silk cocoon fibers. The protein sericin, shown in Figure 4, can be isolated through several techniques, including boiling, high alkali treatment, high-pressure steam heating, direct extraction from MSG, and enzymatic treatment. To remove any residual chemicals, the final product of an aqueous solution of sericin protein with different molecular weights that must be dialyzed against distilled water using cellulose tubing (MWCO 3.5 kDa), with frequent water changes (Kabir et al., 2024; Kunz et al., 2016).

**FIGURE 5:**
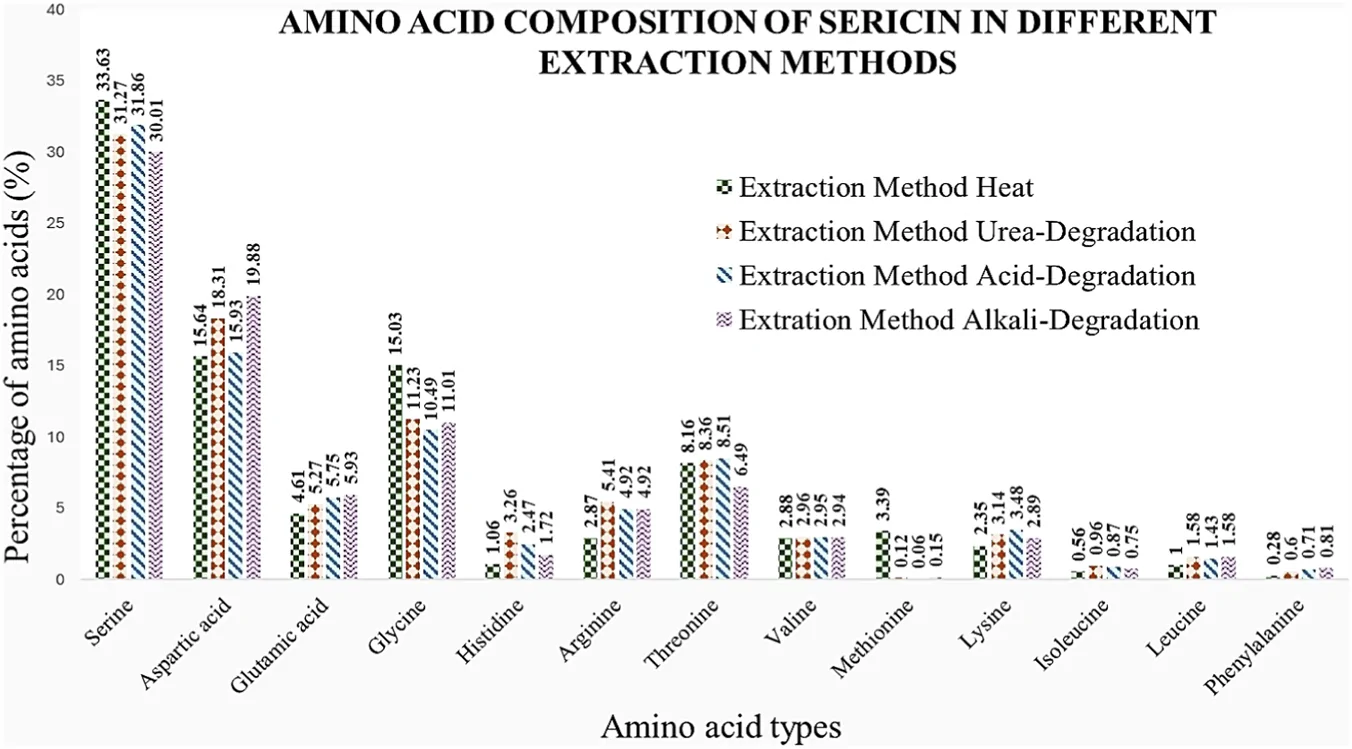
Amino acid composition of sericin in different extraction methods.

Sericin can also be gently extracted from silk filaments through enzyme treatment. Reports indicate three enzymes can remove sericin: i) trypsin, ii) papain, and iii) bacterial enzymes. Acid or alkaline proteases, at doses of 2–2.5 g/L, can be used to extract sericin enzymatically. Additionally, trypsin solutions and enzymatic hydrolysis methods have been employed for sericin extraction at various temperatures and durations to achieve small-peptide sericin hydrolysates with improved solubility and antioxidant activity for applications in food, cosmetic, and pharmaceutical. Typically, 1% trypsin enzyme is maintained for 10 h at 37 °C within a pH range of 7–9 to extract sericin (Hu et al., 2023). The amount of sericin extracted using 1% and 8% trypsin solutions over 4 h was 26.4% and 28.7%, respectively. Using the bacterial enzyme alcalase at 60 °C for 1 h at pH 9 proves more effective than trypsin and papain (Mazurek et al., 2024; Sangwong et al., 2016). The amino acid composition of sericin varies with the extraction method, as illustrated in Figure 5. Boiling or treating silk cocoons with bromelain, a proteolytic enzyme, is a popular, eco-friendly approach to “degum” silk cocoons that produces small sericin peptides (about 5–20 kDa) (Hong et al., 2019). Ionic liquids (ILs) are emerging as green solvents for sericin extraction. They disrupt hydrogen bonds and electrostatic interactions, facilitating sericin removal without damaging silk fibers. Certain ILs, such as tetraethylammonium bromide (TEAB) and 1-Butyl-3-methylimidazolium chloride (BMIM.Cl), have proven effective in extracting sericin, making them suitable, environmentally friendly options (Kalita et al., 2024).

Traditionally, alkaline solutions like sodium carbonate are used for degumming silk. Although this method is effective, it can be time-consuming and energy-intensive (Pandey et al., 2024). The most common material for alkali treatment is sodium carbonate; however, alternatives include caustic soda, trisodium phosphate, sodium silicate, borax, and sodium bicarbonate. This process involves boiling cocoon pieces in a 0.02 M sodium carbonate (alkali) solution for 30 min. The alkali-soluble solution is collected, filtered, and dialyzed. For optimal degumming, surfactants such as sodium lauryl sulfate, alkyl aryl sodium sulfonates, and ethylene oxide condensates, including polyoxyethylene alkyl ethers and polyethylene glycol ethers, are recommended. The urea method involves soaking cocoon pieces in an 8 M urea solution for 30 min, followed by refluxing at 85 °C for another 30 min, then filtration and dialysis (Vleugels et al., 2015).

Acid treatment includes degumming with dilute acids such as citric acid, succinic acid, tartaric acid, and monochloroacetic acid. Acid hydrolyzes sericin protein by targeting the peptide bonds between aspartic and glutamic acids (Zhang et al., 2019) which are found in higher abundance in sericin, making it a selective process. In acid extraction, a 1.25% citric acid solution is used to cut cocoons and is boiled for 30 min. After filtration, the solution undergoes dialysis. Compared to conventional methods, the novel approach combining steam degumming with microwave pre-treatment drastically reduces the time and energy required for sericin extraction. This method maintains silk’s mechanical properties while achieving high degumming efficiency, making it a greener alternative (Saad et al., 2023).

The gum’s solubility in hot water allows various temperature-based methods for gum removal during degumming. One technique involves boiling raw silk in water, spray-drying it, and then dissolving it in distilled water primarily to extract and refine silk sericin, making it a sustainable approach to recover high-quality sericin powder from silk manufacturing wastewater, rather than discarding it. Infrared heating is an another highly efficient, eco-friendly method for sericin extraction from silk waste compared to conventional high-temperature, high-pressure (HTHP) methods uses less water, generates fewer pollutants, shortens treatment times, and operates at lower temperatures Alternatively, small pieces of silk cocoons immersed in deionized water and autoclaved to produce dissolved high molecular weight, and lower molecular weight sericin (Da Silva et al., 2014), which could be stored as a dry powder. In high-pressure steam heating, clean-cut cocoons are autoclaved at 120 °C in a small amount of water, then filtered, frozen, and lyophilized. Furthermore, the extraction of sericin from glands involves removing MSG from mature larvae and cutting it into three parts along the windings. Using forceps, gland cells are carefully extracted from each section. After collecting the gland contents, they are gently shaken for 30 min. The clear, water-soluble supernatant is considered sericin protein from MSG (Hu et al., 2023).

An analysis of various degumming methods for extracting silk sericin concludes that alkali/high-temperature degumming yielded the highest protein content (4.90 g/dL) and is the most efficient. The other effective method includes high-pressure degumming, with protein content yield of 4.58 g/dL.High-molecular-weight (MW) sericin, particularly in the range of 150–330 kDa, is crucial for bioprinting inks due to its properties of viscosity, shear-thinning, and gelation, which ensure a shape fidelity and cell viability greater than 90%. Heating sericin at temperatures between 98 °C–120 °C (boiling or autoclaving) leads to a recovery rate of 26%, but causes hydrolysis of the chains, resulting in a MW range of 15–150 kDa and a decrease in β-sheet structures, thereby impairing printability. Alternatively, treating sericin with hydrochloric acid at pH 4 °C and 80 °C results in excessive degradation, yielding MWs below 50 kDa and limiting bioactivity. In contrast, using urea at 8 M concentration at room temperature to 60 °C for 1–4 h effectively disrupts hydrogen bonds without cleaving peptides, leading to clearer high-MW bands in the 75–250 kDa range, five times the amount of β-sheets compared to heat treatment, and enhanced cell adhesion and proliferation (Saad et al., 2023). Urea-high-MW SS inks display thixotropic properties with a recovery of G′ greater than 80% after shear, simulating the extracellular matrix (ECM) for the creation of vascularized bone scaffolds, resulting in a twofold increase in alkaline phosphatase (ALP) levels. When blended with silk fibroin (SF), these inks resolve 200 μm without swelling. The use of fibroin-deficient cocoons for standardization enhances these characteristics further (Li et al., 2022). Sericin comprises 20%–25% of the silk of the silkworm and is rich in hydrophilic amino acids (∼76%), mostly serine, aspartic acid, and glycine. Various extraction methods highly affect its composition, like in hot water extraction processes, serine (up to 31%) and aspartic acid are achieved. While acid/alkali methods alter molecular weight and yield varying amino acid compositions (50–150 kDa for acid, 15–75 kDa for alkali) (Hu et al., 2023).

## 5 Biomedical application of sericin protein

Silk cocoons are natural polymer proteins, mainly composed of fibroin, with sericin covering 25%–30%. Degumming is essential for the textile industry to remove sericin to enhance fiber sheen, whiteness, smoothness, softness, and dyeability. However, annually, nearly 50,000 tons of sericin are discarded in wastewater during silk degumming, causing environmental issues (Kunz et al., 2016). Though in recent years sericin waste has been documented as a valuable potential material, and innovative approaches to “upcycle” this waste are gaining attention to alleviate environmental impacts and create economic value. Sericin contains 18 amino acids and is a biocompatible, biodegradable, and sustainable biomaterial owing to its corrosion resistance, moisturizing, antioxidant, antibacterial properties, and UV protection action that make it valuable for cosmetic, food packaging, and biomedical applications like tissue engineering and wound healing (Gaviria et al., 2023).

### 5.1 Antibacterial

Silk sericin, a protein derived from silk cocoons, exhibits potential antibacterial properties against foodborne pathogens, including *Escherichia coli* and *L. monocytogenes*, making it a promising candidate for medicinal uses. Sericin could enhance food preservation methods, safety, and shelf life. Thus, sericin is not just an industrial byproduct but a promising natural solution for controlling bacterial contamination in the food industry. Silk sericin has proven effective against both Gram-positive and Gram-negative bacteria, including *Staphylococcus aureus*, *E. coli*, and *Pseudomonas aeruginosa*, with inhibition rates reaching up to 94.77% against *Klebsiella pneumoniae*. *Samia ricini* notably showed the highest inhibitory activity against several bacteria (Kanyora et al., 2024). When combined with standard antibiotics, sericin boosts antibacterial activity, achieving significant inhibition zones against foodborne pathogens like *Salmonella typhimurium* (Seo et al., 2022). Sericin’s antibacterial properties are increasingly beneficial for wound healing applications. It is often incorporated into composite biomaterials containing antimicrobial agents such as metal nanoparticles and natural extracts to enhance long-term antimicrobial effects and facilitate wound healing (Mazurek et al., 2024).

The inhibition activity of micrococcus-type bacteria in the agar diffusion method was observed in *Dok Bua* and *Nang Noi* silk cocoons, particularly in sericin. The zone of inhibition measured 9–12 mm in diameter for both 10 and 20 mg/mL sericin concentrations, with antibacterial effectiveness increasing alongside higher concentrations. A related experiment employed the significant dilution method and disc diffusion assay to assess *Eri* sericin’s antibacterial activity against typical gram-negative (*E. coli*) and gram-positive (*S. aureus*) bacteria models (Mohanty et al., 2025). However, Kaur et al. presented a conflicting view, suggesting that resistance to growth from chemical residues during cocoon component isolation or purification might cause microbial infections. Additionally, gum, embedded crystals, and properly isolated silk fibers free of residues showed no evidence of bacterial resistance. The exact mechanism of sericin’s antibacterial activity remains unknown. Moreover, a study showed sericin hydrogel system during gelation could encapsulate hydrophobic drug ciprofloxacin (CIP) to obtain a multifunctional wound dressing with favorable antimicrobial activity. The fabricated CIP-loaded sericin hydrogel displayed antibacterial activity against both Gram-positive bacteria (*S. aureus*) and Gram-negative bacteria (*P. aeruginosa*) *in vitro*. CIP-loaded sericin hydrogel effectively eliminated bacteria in wounds and supported accelerated wound healing in the *in vivo* studies (Li et al., 2025) Overall, the literature indicates that sericin’s antibacterial properties make it a promising component for developing antimicrobial medical bandages, mouthwashes, antibacterial soaps, toothpastes, and air filter coatings. Sericin has also been found to possess antioxidant, anti-inflammatory, and anti-ageing effects, making it a potential drug candidate for cosmetic and medicinal use. Research demonstrates strong bactericidal effects through various techniques, as shown in Table 2. The antibacterial activity of silk sericin-based nanoparticles (NPs) has attracted significant interest due to their biocompatibility and effectiveness against various pathogens. Silk sericin, a protein derived from silk cocoons, functions as a reducing and capping agent in nanoparticle synthesis, boosting their antibacterial properties. Gold nanoparticles were also synthesized using sericin as a reducing agent, showing effective antibacterial action against six different foodborne pathogenic bacteria (Madan et al., 2026). In contrast, synthesized sericin-capped silver nanoparticles (Se-AgNPs) demonstrated bactericidal capabilities against various bacteria, with smaller nanoparticles exhibiting greater antibacterial activity (Summer et al., 2023). Studies indicate that sericin extracts from different silk sources, including *Argema mimosa*, *G. rufobrunnea*, and *G. postica*, exhibit antibacterial effects against bacteria such as *Bacillus subtilis* and *S. aureus*. The effectiveness of sericin depends on factors like concentration and pH, which can optimize bacterial inhibition. The agar well diffusion method is commonly used to evaluate the antibacterial activity of sericin extracts, highlighting their potential as natural antibacterial agents. The antibacterial properties of silk cocoon extracts, particularly from *Bombyx mori*, have shown promising potential in promoting wound healing. These extracts show significant antimicrobial activity against common pathogens such as *S. aureus*, *Escherichia col*i, and *P. aeruginosa*, which are often involved in wound infections (Tao et al., 2021). The sodium alginate hydrogels, when incorporated with sericin, demonstrated antibacterial effects against various bacteria through irreversibly disrupting bacterial membrane structure that lead to bacterial lysis and death (Saad et al., 2023). The mechanism of action is due to positively charged (polycationic) side groups of sericin that interact with the negatively charged surface of bacterial cell walls. Moreover, lower molecular weight components (<20 kDa) of sericin produced during extraction can directly penetrate bacterial walls and bind to bacterial peptidoglycan inhibiting cell growth (Tao et al., 2021). Furthermore, sericin acted as a natural reducing agent to synthesize *in situ* metal nanoparticles (AgNPs), which significantly strengthened the antibacterial capacity against biofilms within the hydrogel. Sericin nanoparticles derived from sericin extracted from cocoons of *A. mylitta*, were coated with poly-l-lysine to make the surface potential positive has demonstrated antibacterial activity against *S. aureus*, and *E. coli* (Dutta et al., 2020). Silk sericin acquired through high-temperature-high-pressure degumming was combined with polycaprolactone (PCL) to make cross-linker-free hydrogels. The optimal 5:1 (SP5) and 6:1 (SP6) ratios made stable matrices, confirmed by UV-Vis, FTIR, XRD, SEM, and rheological analyses. SP5 showed superior porosity, swelling behavior, and drug encapsulation efficiency and reached a controlled zero-order drug release through Fickian diffusion. These hydrogels exhibited broad-spectrum antibacterial activity against both Gram-positive and Gram-negative bacteria. These biodegradable hydrogels have shown the ability for transdermal drug delivery, especially in wound healing and pain management (Deb et al., 2024).

**TABLE 2:** Effective antibacterial property of silk sericin.

Sericin isolated from	Bacteria studied	Antibacterial assay	Observations	Ref.
*Argema mimosa, G. rufobrunnea, G. postica*	*B*.*subtilis*, *S*.*aureus*, and *Staphylococcus*	Agar well diffusion analysis	Sericin extracts showed antibacterial efficacy dependent on concentration and pH	Manesa et al. (2020)
Cocoons of *A. mylitta*	*S. aureus* and *E. coli*	minimum inhibitory concentration (MIC) and Bacterial Live/Dead assay	The ethanol desolvation method produced sericin nanoparticles that significantly inhibited the growth of Gram-positive bacteria in comparison to Gram-negative bacteria	Dutta et al. (2020)
*Bombyx mori*	*S. aureus*, *E. coli, S. haemolyticus, P. aeruginosa, K. pneumoniae*	Minimum inhibitory concentration (MIC), minimum bactericidal concentration (MBC), Zone of inhibition studies, Growth kinetics, Morphology, Bacteriostatic analysis	Significant bactericidal activity The hydrogels, incorporated with sericin, demonstrated antibacterial properties	Boonpavanitchakul et al. (2020), Shetty et al. (2026), Xue et al. (2016), [68] Tao et al. (2021), Zhang et al. (2021)
Cocoon’s of *Eri* silkworms	*E. coli*, *S. aureus*	Disc diffusion assay, Critical dilution micro method assay	Bacterial membrane dysfunction	Senakoon et al. (2009)

Silk sericin serves as an adaptable platform for the delivery of drugs throughout the biological fields, with nanoparticles (sericin-AgNPs) enabling antibacterial/antifungal activity in nanofibers, sponges, and scaffolds. Hydrogels of chitosan/resveratrol offer antimicrobial, antioxidant, and anti-inflammatory wound healing; microneedles (PDA@Ag/SerMA) combining photothermal regeneration for diabetic ulcers provide antibacterial efficacy against *S. aureus* and *E. coli* under 808 nm near-infrared (NIR) light, enabling efficient diabetic wound repair. Films of (sericin/alginate/aloe vera or agarose/ZnO/AgNPs) show significant antimicrobial activity against both Gram-positive and Gram-negative bacteria, suitable for antibacterial coatings and tissue engineering applications (Das et al., 2021). Copper-sericin (Cu-SER) metal–organic frameworks (MOFs) represent an eco-friendly method to treat osteomyelitis through combining antibacterial properties with bone regeneration. These MOFs utilize sericin as an organic template for deposition, mimicking collagen’s role in bone mineralization (Kundu et al., 2022). Silk sericin demonstrates substantial antibacterial properties, along with superior biocompatibility and minimal toxicity, positioning it as a sustainable alternative or supplement to synthetic drugs, especially in wound healing, cosmetics, and biomedical applications, as shown in Table 2. However, its antibacterial efficacy is generally lower than that of synthetic agents like silver nanoparticles or antibiotics, it may be most effective when used in synergistic formulations to enhance overall effectiveness.

### 5.2 Anticancer properties

Sericin with 18-amino acid is hydrophilic and rich in serine. Its main components are strongly polar side groups. As a glycoprotein, sericin has biochemical properties including immunomodulation, antioxidation, and suppression of tyrosinase and elastase activities (Das et al., 2023). Excessive and prolonged alcohol intake often causes alcoholic liver disease (ALD). Various other mechanisms also contribute to ALD, including the production of reactive oxygen species (ROS) and other free radicals, which occur through processes such as the activation of Kupffer cells, cytochrome P4502E1-mediated ethanol oxidation (Li et al., 2008). Study shows that sericin can reduce reactive oxygen species, protect cells from oxidative damage caused by ultraviolet B (UVB) radiation, inhibit cancer growth, and possess antibacterial properties. Known for its antioxidant qualities, sericin may help fight different types of cancer cells. It inhibits DMBA-TPA-induced effects in skin cells and induces apoptosis in colon cancer cells by increasing caspase-3 activity and decreasing Bcl-2 expression (Kumar et al., 2018). Zhaorigetu et al. (2001) studied sericin’s ability to inhibit tumor growth in colon cells and its relationship to antioxidant activity. Dietary supplementation with sericin significantly reduces tumor incidence and lowered markers of oxidative stress compared to control groups fed with standard casein protein diet (Zhaorigetu et al., 2001). Demonstrated that a high-sericin diet significantly suppresses colon tumor development, with only 1 in 14 mice developing tumors compared to 5 in 14 controls. This suppression is linked to reduced colonic lipid oxidation and a 62% reduction in tumor incidence (Zhaorigetu et al., 2001).

Sericin’s suppressive action during the initiation and promotion stages of colon tumorigenesis may explain these observations. Studies indicate that sericin protects mice from alcohol-induced liver injury. Administration of sericin in mice suggests that sericin accelerates alcohol metabolism, enhances antioxidant defences, and reduces oxidative stress, to prevent damage to liver cells (Chen et al., 2025). Liu et al. (2017) developed silica mesoporous silica nanoparticles (SMSNs) for targeted doxorubicin delivery to drug-resistant cancer cells. The study demonstrates that amphipathic sericin coating stabilizes the MSN platform and provides a capping effect to prevent premature drug release, leading to enhanced cellular uptake of MSN into drug-resistant cancer cells, facilitating entry into perinuclear lysosomes, and triggering apoptosis (Liu et al., 2017). According to Niu et al. (2021), sericin’s anti-cancer effects are mainly mediated by inhibiting the PI3K/Akt signalling pathway, which is crucial for the development and progression of triple-negative breast cancer (TNBC). The studies indicated that sericin inhibits proliferation in TNBC cell lines (MDA-MB-468) while causing little to no toxicity in normal breast epithelial cells (MCF-10A) (Niu et al., 2021). Jolly et al. (2022) identified the p38 MAPK pathway as a key mechanism for cellular stress responses and apoptosis regulation, with sericin activation indicating a signalling cascade that induces programmed cell death in PC3 cells. Results indicated that sericin causes cell death *via* mechanisms different from those of caspase-3 and PARP, signifying the activation of alternative apoptotic pathways that may benefit cancer therapy when conventional pathways are ineffective. Additionally, sericin induces G0/G1 phase cell cycle arrest, thereby inhibiting cancer cell growth and increasing their susceptibility to apoptosis, that is vital for its effectiveness as an anticancer agent (Jolly et al., 2022). Sericin shown to significantly affects the survival of HCT116 colorectal cancer cells, inducing apoptosis in over 80% of the treated population (IC50 = 42.00 ± 0.002 μg/mL). Mechanistically, sericin activates the extrinsic apoptotic pathway through caspase cascades involving CASP8/10 and CASP3/7, as shown by the overexpression of multiple CASP genes, FASLG, and TNFSF10 (Ratanabunyong et al., 2024). Sericin-derived nanocarriers (SNC) efficiently delivered cisplatin (Cispt-SNC) into MCF-7 breast cancer cells, demonstrating the potential of protein-based nanocarriers for targeted cancer therapy (Bahremand et al., 2024). Sericin nanoparticles have been demonstrated to be effective in photothermal therapy due to their efficient heat conversion and prolonged circulation in the bloodstream. They accumulate at tumor sites, improving treatment effectiveness. Combining pH-sensitive chemotherapeutics and photosensitizers, such as doxorubicin and indocyanine green, further enhances therapeutic efficacy by inducing immunogenic cell death (Li X. et al., 2023).

### 5.3 The effect of sericin on reactive oxygen species (ROS)

Sericin, derived from silkworm cocoons, demonstrates significant protective effects against oxidative stress and related cellular damage. It boosts antioxidant enzyme activity, scavenges reactive oxygen species (ROS), and prevents lipid peroxidation, thereby safeguarding cellular structures such as mitochondria and nuclei. Sericin demonstrated protective action against 1,2-dimethylhydrazine (DMH)-induced colon carcinogenesis and UVB-induced skin damage/tumor promotion in mice by reducing oxidative stress (Sasaki et al., 2000; Rahimpour et al., 2023). Sericin acts as a strong antioxidant by reducing levels of 4-hydroxynonenal (4-HNE) a marker of oxidative stress in the epidermis of treated mice and downregulating cyclooxygenase-2 (COX-2) protein expression, a key enzyme involved in inflammation and tumor promotion. This reduction is significant because 4-HNE forms adducts with proteins, contributing to cellular damage and tumor progression (Rahimpour et al., 2023; Zhaorigetu et al., 2003a). Additionally, sericin has displayed notable potential in suppressing tumor formation in mouse skin models induced by DMBA-TPA. This effect is mainly due to its ability to reduce oxidative stress, inflammatory responses, and the expression of tumour necrosis factor-alpha (TNF-α), an endogenous tumour promoter. The decrease in 4-HNE, a byproduct of lipid peroxidation, further supports sericin’s role in mitigating tumorigenesis (Zhaorigetu et al., 2003a). Moreover, 4-HNE, a byproduct of lipid peroxidation, is a specific inducer of COX-2 gene production. COX-2 is an enzyme that catalyzes various reactions during prostanoid formation. As a pro-inflammatory mediator, COX-2 is involved in cell division, skin inflammation, and the development of skin tumours. It is well known that oxidative stress increases COX-2 expression. Topical application of sericin significantly reduces epidermal COX-2 expression. It is suggested that sericin suppresses oxidative stress, thereby decreasing COX-2 levels in the epidermis (Zhaorigetu et al., 2003b). Studies show that topical sericin inhibits the production of c-fos and c-myc, proto-oncogenes linked to various forms of carcinogenesis (Zhaorigetu et al., 2007). ROS is believed to act as important secondary messengers that can induce the expression of immediate early response genes, including c-myc and c-fos. Sericin has demonstrated anti-tumor effects on colon cancer cells by activating caspase-3 and suppressing Bcl-2 expression. Additionally, by regulating the Akt signalling pathway, sericin may protect the hippocampus from damage and delay apoptosis in diabetic rats by reducing hyperglycemia-induced oxidative stress, regulating growth hormone/insulin-like growth factor-1 (GH/IGF-1) axes, and inhibiting neuroinflammation (Chen et al., 2012; Salari et al., 2023). Hyperglycemia in diabetics activates the c-Jun N-terminal kinase pathway, which triggers insulin resistance by inhibiting Akt activation and reducing its phosphorylation (Yung and Giacca, 2020; Zhou et al., 2023). This can damage hippocampal blood capillaries, which can cause regional ischemia, triggering a cascade of cellular death mechanisms, including an increase in the pro-apoptotic protein Bad. In the study, sericin treatment was associated with protective effects by upregulating the Akt signaling pathway, increasing p-Akt expression, and activating NF-κB, Conversely, decreased pro-apoptotic protein Bad expression was observed, suggesting an anti-apoptotic effect of sericin. At a dose within the physiological range, *A. mylitta* silkworm sericin prevents UVB-induced apoptosis in HaCaT cells (Dash et al., 2006; Dash et al., 2008). In UVB-irradiated cells, sericin reduces apoptosis by inhibiting caspase activity and possibly lowering oxidative stress. In related research, sericin from *A. mylitta* silkworm cocoons protected feline skin fibroblasts from hydrogen peroxide-induced oxidative damage. Additionally, a negative correlation was identified between sericin’s antioxidant activity and its molecular size; the greater the molecular size, the greater the antioxidant activity (Wei et al., 2022).

Through targeted modulation of important signaling pathways, silk sericin (SS) exhibits complex anti-inflammatory and antioxidant properties that go beyond simple free radical scavenging to affect cellular redox homeostasis as well as immune responses. Activation of the Nrf2/ARE (Nuclear factor erythroid 2-related factor 2/Antioxidant Response Element) pathway serves as a master regulator of antioxidant defense, involves SS, which promotes the translocation of Nrf2 into the nucleus where it binds to ARE sequences, leading to increased expression of phase II detoxification enzymes like superoxide dismutase (SOD), catalase (CAT), glutathione peroxidase (GPx), and heme oxygenase-1 (HO-1) (Jantaravinid et al., 2024). In studies on H2O2-stressed osteoblasts and macrophages, these enzymes showed a 2–3-fold increase within 6–24 h of SS treatment (0.1–1 mg/mL), significantly reducing reactive oxygen species (ROS) by 40%–60% and restoring mitochondrial membrane potential. This effect is isoform-dependent, with aspartic acid residues in Ser2 enhancing binding affinity to Nrf2, which supports SS’s role in alleviating oxidative stress during bone regeneration (Jantaravinid et al., 2024). Additionally, sericin suppresses NF-κB signaling by preventing IκBα phosphorylation and p65 translocation, thereby inhibiting the transcription of pro-inflammatory cytokines such as TNF-α, IL-6, IL-1β, and iNOS. Experiments with LPS-stimulated RAW 264.7 cells demonstrate that SS (at 0.5%–2%) can dose-dependently reduce cytokine mRNA and protein levels by 50%–80% after 24 h. Its profile resembles that of dexamethasone but lacks its glucocorticoid side effects. This anti-inflammatory effect is linked to the stability provided by Ser1’s β-sheet, which encourages M2 macrophage polarization at implantation sites and enhances biocompatibility (Sun et al., 2022). Moreover, sericin modulates the MAPK Cascade through bidirectional effects on pathways like ERK1/2 (which promotes cell proliferation), JNK, and p38 (associated with stress and apoptosis), depending on the context and SS dose. Low doses (0.1 mg/mL) activate ERK phosphorylation, leading to osteoblast proliferation (evidenced by a 1.5-fold increase in ALP in MC3T3-E1 cells), whereas higher doses (1 mg/mL) suppress JNK/p38 pathways in UV-irradiated keratinocytes, reducing caspase-3 activity by 60%. Hydrogels containing Ser2/3 isoforms are known to maintain MAPK activity balance, which is vital for effective wound healing through regulated apoptosis and ECM remodeling (Kim et al., 2023).

### 5.4 Anti-thrombotic effect

Sulfated sericin influences the blood coagulation cascade, suggesting it has an anti-thrombotic effect. Prothrombin time (PT) and activated partial thromboplastin time (APTT) were found to be prolonged by sulfated sericin. It is observed that sulfated sericin blocks the formation of the usual double-strand fibrils of fibrin monomer (FM) during fibrin fiber production. Additionally, it is shown that sulfated sericin’s anticoagulant action inhibits the early polymerization phase of FM. Sulfated sericin can be produced by condensing salicylic acid, formaldehyde, and sericin in a single step, resulting in a co-polymer with a molecular weight of 6,000–8,000 Da. At concentrations of 0.01–1 mg/mL in blood, it demonstrates anticoagulant, fibrinolytic, and anti-aggregation properties against thrombocytes. It can also be obtained by reacting sericin with chlorosulfonic acid and separating it into three fractions *via* Sephacryl S-200 gel-filtration chromatography. The higher molecular weight fractions of sulfated sericin show greater anticoagulant activity. Its anticoagulant activity is estimated to be one-tenth to one-twentieth that of heparin (Tamada et al., 2004). Sulfated sericin derived from a cocoon has shown to have anticoagulant activity ranging from 1/10 to 1/20 of heparin, according to one study (Tamada et al., 2004). Another study demonstrated that sericin exhibits a strong cardioprotective effect against rat myocardial necrosis and hypertrophy (Jantaravinid et al., 2024; Sun et al., 2022; Tamada et al., 2004; Ahsan et al., 2022). Serum and cardiac tissue analyses showed that sericin therapy increased non-enzymatic antioxidants and decreased oxidative stress markers, while improving several physical, enzymatic, and histological parameters (Ahsan et al., 2022). *In vitro* experiment demonstrates that combining silk fibroin and sericin can be developed into a nano-coatings on medical devices that can improve hemocompatibility. Furthermore, its negatively charged and hydrophilic characteristics can significantly reduce clot formation and platelet adhesion (Miao et al., 2023). Moreover, sericin has anticoagulant properties that help lower the risk of thrombosis by interfering with blood components and modulating the coagulation pathway to prevent thrombus formation (Tamada et al., 2004).

### 5.5 Wound healing effect of sericin protein

Numerous *in vitro*, *in vivo*, and clinical studies have shown that sericin promotes wound healing without provoking inflammation (Mazurek et al., 2024). Research indicates that sericin at 100 μg/mL can stimulate fibroblast L929 migration in *in vitro* scratch tests, which is on par with the positive control with epidermal growth factor at the same concentration. Additionally, in rat models with full-thickness wounds, silk sericin dressings resulted in increased epithelialization and type III collagen production, along with enhanced wound healing (Aramwit et al., 2013a). Collagen formation is vital for wound healing, and sericin demonstrated to boost collagen production significantly. However, studies also show, some strains of sericin are more effective than others (Ersel et al., 2016). However, studies also show, effectiveness of sericin varies on the silkworm strain, the extraction method, and molecular weight of sericin selected. Furthermore, sericin supports the development and adhesion of keratinocytes and fibroblasts in human skin, which is essential for enhanced re-epithelialization for accelerate wound healing (Kamalathevan et al., 2018). Its safety in wound healing products is confirmed by acute cutaneous toxicity tests, which showed no significant adverse effects on the skin. Since SS-based hydrogels and dressings promote cell growth, manage moisture, and possess antibacterial properties, they hold great promise for wound treatment (Arango et al., 2024). Sericin functionalized bacterial cellulose enhanced fibroblast proliferation in one study, suggesting that these materials could benefit tissue engineering and wound healing applications (Lamboni et al., 2016). Moreover, sericin offers a safe and practical approach for treating burn wounds, alongside the first clinical trial involving addition of sericin to silver zinc sulfadiazine cream that demonstrated improved burn treatment, with significantly faster re-epithelialization (Aramwit et al., 2013b). Acute cutaneous toxicity test on rats showed that the cream containing silk protein had no noticeable adverse effects on the skin. Assessment and evaluation of sericin cream and silk protein film for dermal safety, acute dermal toxicity and the determination of irritant and sensitization effects on skin concluded that sericin did not cause an appreciable dermal toxicity in rats. When there is a possibility of a drug being absorbed through the skin, an acute dermal toxicity test can be helpful. According to Padol et al. (2011), it provides information on potential health risks associated with brief skin exposure (Padol et al., 2011).

Compared to wounds treated with a control cream base, rats treated with topical application of 8% sericin cream on full-thickness skin wounds showed reduced inflammation, no ulceration, a greater decrease in wound size, shorter average healing time, and increased collagen production (Aramwit and Sangcakul, 2007). Clinical studies have demonstrated superior results in pain reduction and wound quality compared to conventional dressings like Bactigras®. When bacterial nanocellulose (BNC) wound dressings, incorporated with sericin (for enhanced healing) and polyhexamethylene biguanide (PHMB) (as an antibacterial agent) were used. Study indicated that sericin combined with benzalkonium chloride (BAC) can provide effective, low-toxic antibacterial protection, a preservative system to decrease corneal damage, and enhance cell viability for glaucoma patients requiring long-term anti-glaucoma medications. Hence making it a promising agent for enhancing the safety of conventional preservatives in ophthalmic solutions (Nagai et al., 2013).

Studies using MDA-MB-231 (breast cancer) and Mv1Lu (mink lung epithelial) cells in scratch assays confirmed sericin and fibroin proteins from *Bombyx mori* silk promote wound closure. Sericin promotes the wound healing process by upregulating c-Jun and phosphorylating c-Jun protein. Additionally, phosphorylation of ERK 1/2 and JNK 1/2 kinases was observed. Activation of c-Jun is a result of sericin-induced cell migration, involving the MEK, JNK, and PI3K pathways (Martínez-Mora et al., 2012). Verma et al. (2017) reported the development of a sericin/chitosan-capped silver nanoparticle incorporated in carbopol-based hydrogel for wound care management. The study revealed that the hydrogel is biocompatible, reduces the risk of bacterial infection, is non-irritating, and supports wound healing (Verma et al., 2017). Gilotra et al. (2018) demonstrated that sericin-based nanofibrous mats fabricated using electrospinning technique are biocompatible exhibiting reduced inflammation and promote cell growth proposed to provide a substrate for treatment of chronic wounds like diabetic foot ulcers. Compared to other materials or inflammatory stimulants (like lipopolysaccharide, LPS), these mats, mainly blended with polycaprolactone (PCL) or polyvinyl alcohol (PVA), displayed low cytotoxicity and with no significant *in vivo* immune responses. *In vivo* biocompatibility was confirmed through subcutaneous implantation in mice (Gilotra et al., 2018). Baptista-Silva et al. (2021) utilized peroxidase-mediated cross-linking to develop a transparent sericin-based hydrogel for wound treatment. The hydrogel exhibits outstanding biological activity, antioxidant effects, structural stability, and stretchability. The hydrogel reduces inflammatory reactions and adapts well to wound beds (Baptista-Silva et al., 2021).

### 5.6 Sericin as a supplement in animal cell culture

Sericin, protein, has become a promising supplement in animal cell culture, providing a viable alternative to traditional fetal bovine serum (FBS), offering comparable or superior cell growth. Its use in cell culture media was studied extensively because of its non-immunogenic properties and ability to improve cell viability, proliferation, and adherence. This makes sericin a potential substitute for FBS, and addresses significant limitations of FBS like risks of infections, high costs, ethical concerns and batch-to-batch variations. The benefits of sericin in improving cell culture conditions have been demonstrated in various studies, as shown in Table 3. In several serum-free media, sericin, with a molecular weight ranging from 5 to 100 kDa, has shown to promote cell growth by speeding up the development of hybridoma cells. Sericin hydrolysate has also been shown to protect against serum deprivation in cultured Sf9 insect cells. Repeats of 38 amino acids are thought to prevent serum-deprivation-related death, and decapeptide SGGSSTYGYS, functional partial sequence derived from the 38 residues, found to be effective with enhanced cell viability under serum-free conditions, (Takahashi et al., 2005). Reports has demonstrated that cultured human skin fibroblast attachment and growth have significantly improved by sericin, by mainly the 400 kDa component, after 72 h leading to a 250% rise in cell counts compared to controls without sericin (Tsubouchi et al., 2005). It also accelerates the growth of various mammalian cell lines, such as human hepatoblastoma HepG2, human epithelial HeLa, and human embryonic kidney 293 cells. Additionally, studies have confirmed that sericin significantly improves the proliferation of the murine hybridoma cell line 2E3-O showing effects comparable to bovine serum albumin (BSA) (Terada et al., 2005). Sericin’s antioxidant properties and its ability to act as a nutrient source or growth factor in the absence of serum (FBS) are responsible for these effects. The addition of sericin causes proliferation to occur in a dose-dependent manner. Sericin concentrations of 0.01%–0.1% have been reported to stimulate cells however, 1% sericin may be harmful to the culture. Similar results have been observed with recombinant sericin produced in *Escherichia coli*, a heat-stable alternative to animal-derived serum supplements promotes hybridoma growth showing its mitogenic effect regardless of autoclaving or filtering (Terada et al., 2002).

**TABLE 3:** *In vitro* cell culture studies using silk sericin and their biomedical applications.

Sericin as supplement in biomaterial scaffolds	Cell type	Comments	Ref.
GTA cross-linked collagen/SS membranes	HaCaT, 3T3 mouse fibroblasts	Enhanced cell development, and attachment	Akturk et al. (2011)
SS-poly (vinyl alcohol) scaffolds	L929 fibroblasts from mice, rats, and humans	Increased collagen III synthesis, cell adhesion and proliferation, and *in vivo* wound healing	Xu et al. (2018)
SS-polyacrylamide hydrogels	NIH3T3fibroblast	Tissue regeneration application	Zhang et al. (2020)
3D Matrices of sericin Hope cocoons	Co-cultured human dermal fibroblasts and keratinocytes	biocompatible, non-inflammatory properties, and skin tissue ability	Nayak et al. (2013a)
Gelatin-sericin-laminin cryogels	HUVEC Cell	reduce inflammation, promote collagen deposition	Tyeb et al. (2020)
Silk fibroin/sericin 3D sponges	Human monocytic leukemia cells (THP-1), MG63 cell	Cell attachment and viability observed without toxicity	Siavashani et al. (2020)
PVA/Ser-HAP Membranes	Human periodontal ligament stem cells (hPDLSCs)	Promoted osteogenic differentiation, demonstrated by ALP staining, ARS, and RT-qPCR analysis	Ming et al. (2022)
Silk-based material	Human fibroblast	Excellent cytocompatibility and histocompatibility for skin anti-aging	Su et al. (2019)
Sericin conduit	Schwann cells	Biocompatible, high porosity, and mechanical properties for peripheral nerve regeneration	Xie et al. (2015)
SS microparticles	MSC and nucleus pulposus	Highest proliferation index and inhibit oxidative stress damage, increased secretion of trophic factors that stimulate tissue regeneration	Bari et al. (2023)
Silk sericin hydrogel	Chicken embryo choriallantoic membrane (CAM)	no inflammatory response, promote formation of new blood vessels	Baptista-Silva et al. (2022)

Sericin is widely used as a cryoprotectant in serum-free freezing media, due to its high content of polar amino acids particularly serine (27.3%) it suppresses lipid peroxidation and prevents cellular damage during freezing-thawing processes offers an alternative to FBS, which carries contamination and infection risks. The sericin-based cryopreservation medium has successfully maintained cell viability, attachment and growth after thawing, and have emerged as a highly effective, serum-free, and safer alternative to conventional FBS and DMSO media (Sasaki et al., 2005). The mitogenic effects of sericin are mediated through various signaling pathways, including Src and ERK1/2, depending on the cell type. Furthermore, sericin contains an abundance of hydroxyl amino acids, allowing strong hydrogen bonding and providing a favourable environment for cell attachment and growth, has been proven to support the growth and proliferation of different cell types, including fibrosarcoma cells, L929 fibroblasts, and hybridoma cells, in serum-free media (Sato et al., 2011). In bovine embryo culture, sericin supplementation has been shown to neutralize heat stress effects, enhance blastocyst development, and reduce apoptosis and oxidative stress markers. It can serve as an alternative to FBS, with studies showing embryos cultured with 0.05% sericin have similar development rates and cryosurvival characteristics to those with FBS, while also producing fewer lipid droplets, mimicking *in vivo* conditions (Cao and Zhang, 2015). Silk sericin hydrolysate (SSH) supports the growth of Chinese hamster ovary (CHO) cells and Henrietta Lacks (HeLa) cells, with survival and proliferation rates comparable to or better than FBS media. Notably, SSH at 15.0 μg/mL enhance the expression of growth-related genes, such as CXCL12 in CHO cells and LCN2 in HeLa cells, indicating its potential to promote cell growth and division (Zhang et al., 2019). Low molecular weight sericin has shown biocompatibility, immune response modulation, and wound healing promotion in macrophages and human adipose stem cells (Cherng et al., 2022). Additionally, it was found that sericin promotes flavivirus replication in cell cultures, notably increasing infectious Zika virus particles (Alcalá et al., 2022). Sericin also facilitates cleavage and blastocyst production in bovine embryo culture, resembling *in vivo* blastocysts (Hosoe et al., 2014). Furthermore, at various optimal doses depending on the source, sericin stimulates fibrosarcoma cell development in serum-free culture conditions and enhances nuclear maturation of cow oocytes during collection and *in vitro* maturation without altering DNA fragmentation (Satrio et al., 2022).

## 6 Sericin as a biomaterial for tissue engineering applications

### 6.1 Sericin as a biomaterial

**FIGURE 6:**
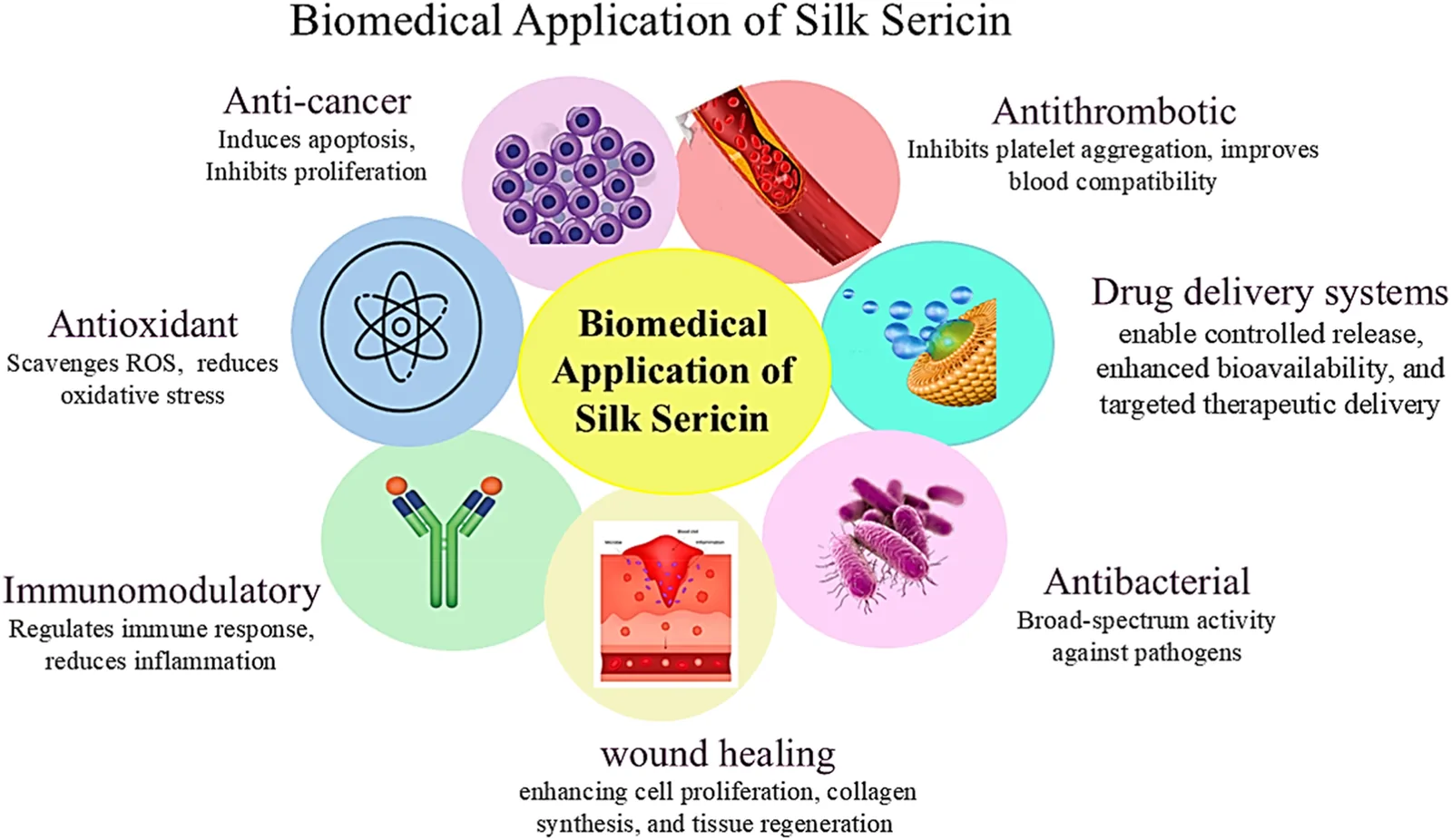
A diagrammatic illustration of the biomedical applications of silk sericin protein.

In tissue engineering, and regenerative medicine, different types of cells, scaffolds, growth-stimulating signals, are the building blocks of engineered tissues, that offer great potential for better patient care, faster healing, and more precise diagnosis. For many years, the focus of biomaterials research has been designing, creating, and improving scaffolds that provide physical support to cells and tissues and serve as delivery routes for drugs and bioactive compounds. Tissue engineering scaffolds have been made using various natural, synthetic, and hybrid polymeric materials to mimic the extracellular matrix of natural tissue. Natural polymers, abundant in nature, such as chitin, fibroin, alginate, starch, collagen, and gelatin, offer promising options mainly because they resemble polymers found in the human body, such as the extracellular matrix (Satchanska et al., 2024). The protein’s versatility is demonstrated by its ability to form various structures, including hydrogels, thin films, and sponges, which can be tailored to meet specific tissue engineering needs. Additionally, sericin’s anti-inflammatory, antibacterial, and antioxidant properties support its use in biomedical applications such as drug delivery and wound healing. A diagrammatic illustration of the biomedical applications of silk sericin protein is shown in Figure 6.

Sericin, due to its excellent biocompatibility, biodegradability, and low immunogenicity offers numerous biological properties is a promising, sustainable biomaterial for tissue engineering and regenerative medicine. Due to its high serine content and ability to support cell adhesion, proliferation, and differentiation, it is fabricated into scaffolds, hydrogels, and films that promote tissue healing and drug delivery. It can be used in tissue engineering and healthcare applications, but requires reprocessing. Sericin is often blended with polymers like gelatin or PVA for improved stability in biomedical applications such as wound dressing. Studies demonstrated that the two main silk proteins, sericin (SS) and silk fibroin (SF), increase the paracrine characteristics of mesenchymal stem cells (MSCs) and trigger extensive cellular responses (Zhang Y. et al., 2023). It has also been shown that SF and SS enhance skin regeneration by regulating the actions of several resident cells in the skin wound microenvironment (Zhang Y. et al., 2023). The properties of sericin scaffolds have been improved through ethanol post-treatments, which have improved their water content, mechanical strength, and crystallinity. Genetic variation is one of the major factors that influence the physicochemical and biological properties of sericin isoforms, and it directly impacts their use in biomedical areas. Major sericin genes Ser1, Ser2, and Ser3 encode the key sericin fractions in *Bombyx mori*. These fractions differ in terms of molecular weight, amino acid composition, solubility, and structure. Whereas Ser2 and Ser3 yield more hydrophilic, readily extractable forms with distinct bioactivities, Ser1 has been characterized by including a large molecular weight, which enables it to form good films and scaffolds. Other genes Ser4, Ser5, and Ser6 were just identified, suggesting that sericin is not a single protein, but a multitude of isoforms, each of which can have distinct biological functions (Wang Y. et al., 2026; Dong et al., 2019; Wu et al., 2024).

### 6.2 Films

Sericin films have become promising biomaterials due to their unique properties and potential uses in various fields, especially in biomedical and pharmaceutical areas. Dimethylolurea (DMU) a crosslinking agent used to produce sericin films with strong tensile strength and high elongation capacity. These films exhibited moderate water vapor permeability, which is useful for applications that need controlled moisture transfer (Turbiani et al., 2011; Joshi et al., 2015). Silk sericin/poly (vinyl alcohol) PVA composite films, especially when crosslinked with genipin, have favorable properties for use as biomaterials in tissue engineering with improved mechanical stability and controlled release (Siritientong and Aramwit, 2012). Adding AgNPs into sericin/PVA composite films, using polydopamine as a reducing agent, has demonstrated long-lasting antibacterial activity, indicating their potential to prevent bacterial growth on medical devices (Cai et al., 2017a). AgNPs attached to the sericin matrix release silver ions (Ag^+^) that disrupt bacterial cell walls, induce membrane damage, and produce reactive oxygen species (ROS), leading to bacterial death. While sericin enhances the stability of AgNPs, facilitates cell proliferation (fibroblasts), and provides a biocompatible matrix for wound healing, amplifying the overall efficacy of the composite. These films are suitable for medical use, as they have shown strong antibacterial effects against common pathogens such as *Staphylococcus aureus* and *Escherichia coli* (Wang Y. et al., 2018). The mechanical strength and water-retention qualities of sericin films are improved by adding polyethylene glycol (PEG) and polyvinyl alcohol (PVA), glycerol, or xanthan gum. These additives, particularly in combination with specific treatment methods, enhance the properties of the inherently brittle sericin films (Zhang et al., 2011; Aramwit et al., 2012; Sothornvit and Chollakup, 2009).

Sericin/collagen membranes displayed improved water-swelling tendency showing beneficial effect as wound dressings when the sericin content is less than or equal to the collagen amount. This allows the membrane to absorb wound exudate and maintain a moist, healing-conducive environment promoting cell attachment and proliferation (Dinescu et al., 2013). Different sericin extraction methods significantly influence the molecular weight, amino acid composition, and secondary structure of sericin hence influence their chemical structure and properties. These variations directly affect the resulting films’ mechanical strength, stability, and bioactivity, such as biocompatibility and antioxidant properties (Jo et al., 2020). Studies have shown that ethanol post-treatments improve the mechanical strength, water stability, and crystallinity of sericin films. These treatments cause morphological and conformational changes in films, making them more suitable for wound regeneration (Arango et al., 2023).

### 6.3 Porous sponges

In tissue engineering, researchers have explored using sericin, a silk protein, as a porous sponge. Sericin’s hydrophilicity, biodegradability, and biocompatibility make it suitable for biomedical applications. A porous three-dimensional scaffold provides several advantages in tissue engineering, as it creates a structure for cells to attach, multiply, and produce extracellular matrix. Reports indicate that human adipose-derived stem cells (hADSCs) have been tested for soft tissue regeneration and wound healing applications on a scaffold composed of 60% collagen and 40% sericin (Dinescu et al., 2013). The research indicates a spongy hydrogel can be created from aqueous sericin using a freeze-thaw process and it offers a “green” alternative, as it is without the use of harsh chemicals. In hybrid collagen-sericin sponges, sericin acts as a cell adhesive protein, and facilitates better cell adhesion and lead to higher proliferation rates of cultured cells. The fabrication process, allows the protein to form a stable 3D network through non-covalent interactions, for developing environmentally friendly and non-toxic biomaterials (Dinescu et al., 2013; Zhang H. et al., 2008). The presence of sericin upregulates expression of the adipogenic marker, peroxisome proliferator-activated receptor γ2 (PPARγ2) in hADSCs. This In turn triggers the upregulation of crucial adipogenic markers: Perilipin, FAS (Fatty Acid Synthase) and aP2 (Adipocyte protein 2) (Dinescu et al., 2013).

Reports have shown that three-dimensional sericin scaffolds containing PVA and genipin (a crosslinking agent) can facilitate rapid healing of full-thickness wounds. Ethyl alcohol (EtoH)-based sericin/polyvinyl alcohol (PVA) scaffolds have also been reported to promote wound healing (Siritienthong et al., 2012). Studies indicate that sericin can be used to produce antibacterial sponges for wound dressings. One study found that sericin interacts with ZnONPs, altering the protein’s structure. FTIR analysis revealed an increase in β-sheet content and a decrease in α-helix and random coil structures. These structural changes may enhance the sponge’s ability to interact with bacterial cells, thereby increasing its antibacterial efficacy (Ai et al., 2019). Song et al. (2024) identified maleylated silk sericin (MSS) and thiolated decellularized heart valve (DHV), which were successfully linked *via* a Michael addition reaction to create a unique sericin covalently modified valve (SCMV). Their study showed that MSS activates endothelial cells (ECs), increasing phosphorylated myosin light chain 2 (MLC2), rho-associated protein kinase 2 (ROCK2), and RhoA expression. These factors support actin polymerization and enhance cell adhesion. Several integrin subunits (α1, α2, α3, α5, α6, β1, β3, and β5) were also involved in this regulation, offering insight into how silk sericin influences ECs behavior (Song et al., 2024). Sericin is extracted and processed to develop products that promote tissue regeneration by providing a biocompatible, bioactive, and biodegradable framework, aiding tissue repair and cell proliferation (Prakash et al., 2024). Additionally, the potential of sericin-based sponges in regenerating bone, cartilage, and nerve tissues has been investigated. The porous nature of sericin sponges provides high porosity, water absorption capacity, and excellent hydrophilicity. Furthermore, sericin sponges have demonstrated strong cytocompatibility with cells. Overall, sericin-based sponges are promising biomaterials for tissue engineering, especially in wound healing and regenerative medicine (Lamboni et al., 2015).

### 6.4 Hydrogels

Hydrogels made of poly (N-isopropylacrylamide) (polyNIPAM) prepared *via* a semi-interpenetrating network (semi-IPN) method, with varying concentrations of silk sericin (SS) have been reported. SS chains dispersed uniformly into the hydrogel polyNIPAM matrix, preventing macro-phase separation (Zhang et al., 2014). The strong hydrogen bonding between the sericin polypeptide chains and the polyNIPAM chains cause the homogeneous distribution in the hydrogels. In hydrogels of poly (N-isopropylacrylamide) (PNIPAm) and SS natural biocompatible genipin (GNP) were used as a cross-linking mediator, to control the release of silk sericin (SS) (Zhang et al., 2014). Without the treatment of trypsin or ethylenediaminetetraacetic acid L929 cells could spontaneously detach from the surface of hydrogels Due to reversible change between hydrophobicity and hydrophilicity, by lowering temperature to 4 °C from 37 °C. In fields like tissue regeneration, cancer or disease therapy, the fabricated hydrogel and separated cells have potential applications. Study have shown the development of *in situ* reconstructive dermal sealants of semi-interpenetrating network of sericin derived from non-mulberry tasar (*Antheraea mylitta*) cocoon and polyacrylamide for treating dermal defects (Kundu and Kundu, 2012). Though in a study, it was reported, to create a biopolymeric matrix (often in the form of a hydrogel) from silk sericin isolated from the cocoons of the tropical tasar silkworm *A. mylitta*, produced through chemical cross-linking with poly (vinyl alcohol). PVA and sericin peptides are chemically modified with methacrylate groups to produce a covalent PVA/sericin hydrogel (Lim et al., 2012). The resulting biopolymeric matrix combines the properties of sericin like high water solubility, biocompatibility, and non-toxicity with the stability of PVA. Sericin positively impacts animal cell culture by enhancing cell adherence and spreading overcoming the inherently poor cell adhesion properties of PVA surface. Hydrogels based on sericin demonstrated great potential for accelerating wound healing process when combined with plant extracts (like banana peel powder, curcumin, or guava leaf) and silver nanoparticles (Munir et al., 2023). Furthermore, secondary polymer like PVA or chitosan, often added to enhanced mechanical strength, provided a synergistic mechanism to speed up tissue regeneration (Ekasurya et al., 2023). Investigations reported significantly reduced pro-inflammatory markers, such as interleukin-6 (IL-6), and increase anti-inflammatory cytokines, such as IL-10 in sericin hydrogels. Subsequently directing a fast transition from the inflammatory phase to the tissue regeneration phase. By modulating inflammatory cytokines and boosting antioxidant enzyme levels (glutathione (GSH), glutathione peroxidase (GPx), catalase (CAT), and superoxide dismutase (SOD) these hydrogels alleviate oxidative stress in the wound bed, by reducing malondialdehyde (MDA) levels with enhanced wound contraction and shorten healing time (Munir et al., 2023).

The sericin-hope silkworm, a specially bred mutant, produces cocoons composed of nearly 98.5%–99% sericin, used to produce elastic, biocompatible, and self-gelling hydrogels, often without chemical crosslinkers, through methods like adding ethanol or autoclaving at 110 °C (Teramoto et al., 2005) Silk sericin (SS) based IPN hydrogels, specifically paired with poly (methacrylic acid) (PMAA) for pH-responsiveness and poly (N-isopropylacrylamide) (PNIPAM) for thermo-sensitivity, have been investigated for stimuli-responsive drug delivery systems. These hydrogels utilize the natural protein sericin for control release of bovine serum albumin (BSA). The pulsatile release behavior of IPN hydrogels suggests they can be formed into thermo-valves or microcapsules that act as on-and-off release controllers (Zhang et al., 2014; Ilyas et al., 2025). Studies demonstrate that sericin-based hydrogels can transfer retinal pigment epithelium (RPE) cells, promoting their proliferation and differentiation (Kim et al., 2022). Additionally, osteogenic factors and osteoblastic progenitors have been encapsulated in sericin hydrogels to support bone tissue engineering (Zhang et al., 2022). Because sericin hydrogels are cytocompatible and promote cell growth, they are also explored for skin tissue regeneration and wound healing. Furthermore, the application of sericin hydrogels in tissue regeneration across various organ including the cornea, bone, skin, teeth, cartilage, and eardrum has been examined (Arango et al., 2021).

### 6.5 Bio coatings

Silk sericin, a hydrophilic protein, has garnered interest in tissue engineering and bio-coating applications due to its biocompatibility, biodegradability, and bioactivity. Sericin both with a β-sheet structure and a significant molecular weight, can effectively induce hydroxyapatite nucleation (Takeuchi et al., 2005). Titanium surfaces modified by poly (methacrylic acid) (P (MAA)) and subsequently immobilized with silk sericin (Zhang F. et al., 2008). Sericin immobilized surfaces display enhance osseointegration, promote osteoblast adhesion, proliferation, and with increased alkaline phosphatase activity, simultaneously showing antibacterial action (Zhang F. et al., 2008; Nayak et al., 2013b). Cellulose fiber surface modified with silk sericin lead to improved functional properties (Kongdee et al., 2005). The presence of sericin improved moisture regain and water retention properties. Due to the increased functional groups treated fabrics show higher color strength and deeper shades when dyed with acidic dyes. Introduce anti-inflammatory, antimicrobial, and UV-protective properties, making the cotton suitable for functional, eco-friendly textiles. Sericin was effectively combined with poly (ε-caprolactone) (PCL) scaffolds, that have improved osteogenic development of mesenchymal stem cells and increased their hydrophilicity (Iranpour et al., 2022). Furthermore, sericin’s capacity to stimulate the paracrine activity of mesenchymal stem cells through integrin and glycolytic pathways highlights its potential for advancing skin wound healing and regulating resident cell behavior in wound conditions (Zhang Y. et al., 2023). The incorporation of sericin into composite scaffolds, including poly (lactic acid-co-glycolic acid) (PLGA), has shown promising effects in treating periodontal disease by providing sustained anti-inflammatory action while also promoting cell adhesion and proliferation (Chachlioutaki et al., 2022). Sericin’s broad applications and its non-cytotoxicity ability promotes tissue regeneration, underscore its significant potential as a biocoating material in tissue engineering (Mohanty et al., 2025).

### 6.6 Micro-nano structures

Research suggested sericin–alginate–chitosan microcapsules microcapsule with an outer chitosan shell and a core of sericin-alginate establish a highly effective, biocompatible model for live cell encapsulation, primarily for hepatocyte transplantation and liver support (Nayak et al., 2014). The sericin-alginate core supports high cell viability and metabolic activity, while the chitosan shell provides structural stability. The capsules, cross-linked with genipin, shows increased resistance and limited swelling, making more stable compared to conventional alginate capsules. Investigation describes development of self-assembled silk sericin/poloxamer (SS-P) nanoparticles with a size range of 100–110 nm tailored to deliver both hydrophilic and hydrophobic drugs (Mandal and Kundu, 2009). *In vitro* studies on MCF-7 breast cancer cells exhibited efficient internalization of SS-P nanoparticles, suggesting high bioavailability at the tumor site indicating their potential for targeted cancer therapy. SS-P nanoparticles loaded with paclitaxel effectively reduced the viability of MCF-7 cells by inducing apoptosis, validated by Annexin V staining and fluorescence microscopy. Sericin/chitosan-capped silver nanoparticle (S/C-SNP) loaded hydrogel formulation showed strong antibacterial activity and facilitated wound closure, preventing infection and speeding up healing addresses restrictions of conventional silver-based antiseptics. Superior bactericidal activity against pathogens such as *S. aureus* and *E. coli* was demonstrated by optimized hydrogel formulation (sericin/chitosan nanoparticle. *In vivo* studies in rats verified its biocompatibility and non-irritating nature, ensuring effective healing (Verma et al., 2017). Sericin nanoparticles have been used to improve controlled release and targeted delivery of both hydrophilic and hydrophobic drugs specifically due to their amphipathic nature (Ma et al., 2025). In mesenchymal stem/stromal cells (MSCs) oxidative stress leads to senescence, aging, and reduced therapeutic function. Antioxidant-loaded SNPs (loaded with proanthocyanidins, quercetin, or epigallocatechin gallate) defend MSCs from oxidative stress, fostering tissue regeneration (Orlandi et al., 2020). Similarly, study displays sericin nanoparticles loaded with polyphenols serve as effective, slow-release delivery systems protects mesenchymal stem cells (MSCs) from oxidative damage. These nanoparticles as antioxidant carrier increase MSCs metabolic activity and longevity under oxidative stress promoted tissue regeneration (Orlandi et al., 2020). Sericin nano-gel clumps have been shown to trigger mesenchymal stem cells (MSCs) to form cartilage-like micro-tissues without needing external growth factors (Zhang Q. et al., 2023). This occurs as the nano-gels mimics soft, and hydrated environment of the pericellular matrix (PCM), that surrounds chondrocytes creating a mechanical environment to promote MSCs chondrogenesis (Zhang Q. et al., 2023). The nano-gels are thought to enable self-assembly and agglomerates MSCs into 3D spheroids, forming a structural linker between cells. Henceforth, sericin agglomerates generated mechanical environment triggers signaling pathways (TGF-β) associated with cartilage development without any external stimuli requirement. Increased glycosaminoglycans and collagen type II, key components of functional cartilage were specified in the micro-tissues produced. Sericin-mediated gold nanoparticles (SSP-AuNPs) because of their strong antibacterial and antioxidant effects have demonstrated exhibited significant, concentration-dependent properties, achieving roughly 70%–71% closure rates equivalent to conventional treatments (Das et al., 2023). Additionally the particles displayed strong scavenger potential against free radicals, vital for reducing wound inflammation. Table 4, shows the list of silk sericin-based biomaterials and their biomedical applications.

**TABLE 4:** Silk sericin-based biomaterials and their biomedical applications.

Forms of sericin	Application	References
Films		
Sericin gel film/membrane	Wound dressing	Padol et al. (2011), Jaramillo-Quiceno et al. (2023), Khanapure et al. (2023), Liu et al. (2022)
Sericin-gelatin	Biopolymeric graft material	Lee et al. (2023)
Ag/ZnO/sericin/agarose	Antimicrobial applications	Li et al. (2021)
Sericin-hydroxyapatite based membranes	Osteogenic activity	Ming et al. (2022)
Chitosan-silver-sericin films loaded with moxifloxacin	Antibacterial potential	Shah et al. (2019)
Sericin-PVA, sericin-curcumin-loaded alginate/PVA	Wound dressing, drug delivery	Siritientong and Aramwit (2012), Cai et al. (2017a), He et al. (2017), Yamdech et al. (2024), Cai et al. (2017b)
Self-assembling fibroin/sericin films	Skin tissue engineering	Byram et al. (2024)
AgNPs/sericin/agar film	Antibacterial capability, wound healing	Wang et al. (2018a), Tyeb et al. (2018), Liu et al. (2018)
Sericin films reinforced with bamboo-derived cellulose nanofibrils	Food packaging, drug delivery carriers, and in wound dressing materials	Nam et al. (2021), Kwak et al. (2018)
Sericin and keratoses	​	Caringella et al. (2022)
Sericin-collagen	Wound dressing	Dinescu et al. (2013)
Sericin-chitosan, ricestarch/chitosan/sericin	Food and pharmaceutics, wound healing and antibacterial, thin films	Nguyen et al. (2025), Rosas et al. (2024), Tachai et al. (2025)
Polyhexamethylene biguanide (PHMB)-sericin	Wound dressing	Lu et al. (2023)
Silk-based composite films	Skin-attachable electronics	Byram et al. (2024), Padol et al. (2011), Sothornvit and Chollakup (2009), Wongtechanon et al. (2025)
Graphene oxide/silk sericin/carbon nanotube	Real-time humidity-sensing	Lee et al. (2025)
Implantable sericin film	Cardiovascular electronic devices	Lv et al. (2025)
Pectin/sericin	food packaging, anti-inflammatory and anti-angiogenic properties	Nguyen et al. (2026), Buabeid et al. (2022)
Nanopatterned films of silk sericin and gellan gum	Photonic applications	Torres et al. (2025)
Sericin and xanthan gum	Biomedical applications	Kumar et al. (2025)
Sericin/natural rubber/chitin whisker film	Elasto-gel dressing	Watthanaphanit and Rujiravanit (2017)
3D-sponges		
Silk fibroin/sericin sponge	Skin tissue engineering, vascular repair, nerve scaffolds	Boonpavanitchakul et al. (2020), Siavashani et al. (2020), Arango et al. (2023), Yao et al. (2024)
Sericin- collagen	Wound healing, cartilage tissue engineering	Lee et al. (2023), Sleiman et al. (2024)
Sericin Hope matrices	Wound dressing	Nayak et al. (2013a)
Chitosan-sericin-halloysite (CMC/Ser-Ag/HNT), Chitosan/sericin porous scaffolds	Antibacterial and wound dressing	Li et al. (2023b), Karahaliloglu et al. (2017)
Sericin/carboxymethylcellulose/polyvinyl alcohol, Sericin-carboxymethyl	Drug delivery, Wound dressing	Boonpavanitchakul et al. (2020), Jaramillo-Quiceno et al. (2023), Nayak and Kundu (2014)
Sericin- PVA	Wound healing	Ai et al. (2019), Arango et al. (2023)
Cellulose/poly (vinyl alcohol)-based silk sericin and azithromycin	Wound dressing biomaterial	Bakadia et al. (2022)
CNT/Sericin Nerve Guidance Conduit, sericin/silicone nerve guidance conduit	Repair of transected peripheral nerves	Li et al. (2020), Xie et al. (2015)
Hydrogels		
FGF1 fabricated sericin hydrogel	Tissue and medical engineering	Wang et al. (2018b)
Silk-reinforced collagen hydrogels, Collagen/Hyaluronan/Sericin	3D culture, Controlled drug release	Sanz-Fraile et al. (2020), Vulpe et al. (2018)
sericin/gelatin methacrylate crosslinked hydrogel	Accelerated wound healing	Chen et al. (2025)
Gelatin carrageenan sericin hydrogel	Bone repair and regeneration	Ashe et al. (2020)
Sericin/Nano-Hydroxyapatite- Graphene Oxide, hymosin beta 10 loaded ZIF-8/sericin hydrogel	Bone regeneration, angiogenesis	Fu et al. (2023a), Gao et al. (2024)
SS-CMC-PVA hydrogel films, Gelatin-Silk Sericin Incorporated with Poly (vinyl alcohol)	Wound healing and drug delivery, articular cartilage defects repair	Jaramillo-Quiceno et al. (2023), hao et al. (2021)
Sericin/polyacrylamide	Reconstructive dermal sealant	Kundu and Kundu (2012)
Fibroin (SF)/sericin (SS) nanocomplex	Tumor modulation and treatment	Gou et al. (2023)
Sericin hydrogel delivering human lactoferrin	Cancer treatment	Xu et al. (2021), Hassan et al. (2024)
Poly (γ-glutamic acid)/silk sericin (γ-PGA/SS) hydrogel	Biomedical and clinical	Shi et al. (2015)
SS-PVA-Na_3_Cit hydrogel, SS-PVA	Flexible, stretchable and conductive hydrogel	Wang et al. (2022), Fu et al. (2023b)
sericin-alginate-chitosan microcapsules, sodium alginate (SA) and silk sericin (SS), sericin-alginate	hepatocytes encapsulation for enhanced cellular functions, cartilage defect repair, oral administration of insulin	Nayak et al. (2014), Faiz et al. (2026)
Silk sericin-based hydrogel with plant extracts	Wound dressing	Zahoor et al. (2023)
Photo-crosslinked sericin urethane methacryloyl hydrogel	Encapsulation and delivery of stem cells	Kader and Jabbari (2022)
Sericin enriched nanocomposite hydrogels	Soft tissue engineering	Șerban et al. (2022)
Sericin/wool keratin	Muscle Tissue Engineering	Demiray et al. (2025)
Poly (2-hydroxyethyl methacrylate) (PHEMA) and silk sericin (SS)	Tissue engineering applications	Tuancharoensri et al. (2023)
methacrylic anhydride modified sericin- royal jelly-derived extracellular vesicles (RJ-EVs)	Accelerate wound healing	Tan et al. (2023)
pH-responsive sericin-pyrrole hydrogel	Antibacterial and antioxidant activities	Wang et al. (2026b)
Nano-fibres		
sericin nanofibers	wound dressing	Dragojlov et al. (2025)
Nitrofurazone-loaded, PLLA-sericin nanofibrers	Wound dressing applications	Zhao et al. (2015)
PS^3D^/TA/SS	regenerative medicine	​
PVA/Sericin, PVA/Sericin/Chitosan nanofibrous	​	Gundhavi Devi and Arun Karthick (2024), Arun et al. (2023)
Sericin and keratin in gelatin based nanofibers	tissue engineering	Vineis et al. (2021)
Coatings		
Titanium surfaces, Tannic acid-assisted deposition of silk sericin on the titanium surfaces	Osseointegration of titanium surfaces	Nayak et al. (2013b), Cheng et al. (2020)
Sericin functionalized with carboxy drugs	Dermocosmetic Applications	Argenziano et al. (2026)
Sericin-coated thin polymeric films (ScF) fabricated from silk fibroin	anti-psoriatic agents	Aramwit et al. (2023)
Coating of polyester fabric	Surface coating properties and durability	Hakeim et al. (2022)
Graphene nanoplatelets/sericine, coated titanium-based alloy	growth and osteogenic differentiation	Mitran et al. (2018)
Nanoparticles		
Sericin-coated silver nanoparticles	Anti-bacterial activity	Summer et al. (2023), Mumtaz et al. (2023)
Self-assembled sericin nanoparticles	Drug delivery and biomedical application	Zhu et al. (2025), Kumar et al. (2022), Cho et al. (2003)
Sericin-poloxamer nanoparticles	Drug or gene delivery	Mandal and Kundu (2009)
Sericin/crocetin micro/nanoparticle	Active drug delivery	Bari et al. (2023)
Sericin nanoparticles loaded with curcumin	Biomedical application	Kanoujia et al. (2021)

## 7 Challenges in using sericin as a biomaterial

Several *in vitro* and *in vivo* studies have demonstrated excellent biocompatibility and diverse biological properties; however, some key challenges remain: i) a reliable and reproducible method for isolating intact sericin protein, ii) instability due to uncontrolled dissolution, iii) low mechanical strength in polymer scaffolds, and iv) sterility issues that need to be addressed in various implant applications. Sericin is traditionally considered a byproduct in silk processing, leading to limited research on efficient extraction methods. The extraction process often involves harsh chemicals, which can compromise the protein’s integrity and functionality (Mazurek et al., 2024). While sericin is generally biocompatible, its immunogenicity can vary depending on the source and processing methods. Ensuring consistent biocompatibility across different batches remains a challenge (Aad et al., 2024). Obtaining intact sericin, and handling of incorporated growth factors (GFs) with poor stability is a critical challenge for sericin-based biomaterials. Moreover, functionalizing sericin to enhance its properties, such as adding growth factors or other bioactive molecules, demands sophisticated methods to maintain stability and bioactivity (Lian et al., 2022). Its hydrophilic nature can cause rapid degradation and loss of mechanical properties, limiting its use in long-term applications (Ma et al., 2025). Sericin lacks the mechanical strength when compared to silk fibroin, which can restrict its use in applications requiring durable materials. Blending it with other polymer materials (PVA, chitosan, gelatine, alginate, etc.) is often necessary to improve its mechanical performance (Silva et al., 2022). The risk of contamination during extraction and processing necessitates strict purification protocols. Despite these challenges, sericin’s potential as a biomaterial remains significant, especially in wound healing and tissue engineering. Its natural properties, including antioxidant and antibacterial activities, make it a promising candidate for developing sustainable and effective biomaterials (Mohanty et al., 2025). However, overcoming challenges related to extraction, stability, and functionalization is essential for expanding its use in the biomedical field.

### 7.1 Sericin immunogenicity and toxicity

Sericin has demonstrated potential as a material in tissue engineering and drug delivery, making this protein an attractive candidate for biomedical applications. However, evaluating its interaction with the host immune system and its biocompatibility is crucial before long-term *in vivo* use. Although many studies show that silk sericin is suitable for biomedical and cosmetic purposes (Fu et al., 2022) some reports discuss immune responses to silk sericin. Nonetheless, research indicates that sericin triggers an immune response when combined with fibroin, while sericin alone shows no immunogenicity (Ode Boni et al., 2022). Several studies emphasize the benefits of silk sericin in wound healing, as it improves the attachment and growth of fibroblasts and human and mouse hybridomas when added to culture media. Additionally, sericin promotes wound healing in rats by stimulating collagen production (Ersel et al., 2016). The levels of inflammatory mediators, IL-1β and TNF-α, are significantly lower in sericin-treated wounds in rat tissues following injury. *In vivo* investigations also reveal that silk sericin peptides do not provoke immune responses (Rahimpour et al., 2023). These results support the *in vivo* use of sericin-based biomaterials. Membranes composed of sericin and sericin gelatin blends have shown good cytocompatibility for tissue engineering applications. Similarly, sericin-PVA scaffolds used *in vivo* demonstrated improved wound healing with reduced scar formation in full-thickness skin wounds, along with lower inflammatory reactions (Siritientong and Aramwit, 2012). Sericin has been shown to exhibit negligible allergenicity and low immunogenicity *in vivo*, supporting its biosafety as a natural biomaterial (Ode Boni et al., 2022). While sericin itself is not highly immunogenic, composites with silk fibroin may induce chronic immune responses due to exposing hydrophobic regions, which can be modulated by coating with hydrophilic polymers (Tian et al., 2025). Studies have shown that sericin is non-mutagenic and nongenotoxic in both *in vitro* and *in vivo* models. A 90-day sub-chronic toxicity study in rats determined a no-observed-adverse-effect level (NOAEL) of 1 g/kg/day, indicating low toxicity under experimental conditions (Qin et al., 2020). In mice, sericin was found to be safe at doses up to 2,000 mg/kg in acute toxicity studies. However, sub-acute studies showed inflammation in vital organs at the highest dose, indicating a NOAEL below 2,000 mg/kg. Sericin’s biocompatibility and bioactivity make it an excellent candidate for tissue engineering and drug delivery systems. It has been used in scaffolds that do not trigger significant inflammatory responses. Silk sericin (SS) is known for its biocompatibility and lack of immunogenicity when pure; however, the presence of residual silk fibroin (SF) due to incomplete degumming can trigger chronic inflammation, which poses significant challenges for its clinical use. Research indicates that while pure SS does not activate a notable immune response in murine macrophages, SF particles and SS-SF composites lead to heightened immune responses, such as the liberation of TNF-α. Insoluble SF has been shown to induce TNF-α surges, whereas SS-coated SF enhances macrophage activation initiated by lipopolysaccharides (LPS) by exposing hydrophobic β-sheet structures. It has been observed that SS-SF scaffolds attract inflammatory cells over extended periods, facilitated by the adsorption of complement proteins onto the fibroin heavy chain (390 kDa), triggering Th2-biased hypersensitivity responses characterized by increased IgE levels and eosinophil recruitment. Furthermore, glycoprotein impurities in SS can aggravate the situation, particularly when obtained from SF, although SS itself appears to suppress interferon-gamma (IFN-γ) without impacting interleukin-10 (IL-10) levels.

The mechanisms behind immune activation linked to SF involve the creation of protein-adsorbing surfaces *via* its crystalline β-sheets, stimulating pathways such as the NLRP3 inflammasome and NF-κB through MyD88, which is in stark contrast to SS’s hydrophilic properties that resist biofouling. Animal studies have depicted that SS hydrogels contaminated with SF lead to granuloma formation, while pure SS does not cause fibrosis. The degree of extraction harshness, like high-heat boiling, may leave behind 1%–5% residual SF, aggravating inflammatory outcomes. (Kunz et al., 2016; Thurber et al., 2015). In relation to clinical translation, the inconsistencies in immunogenicity hinder regulatory approvals; the FDA categorises silk as a device material, but SS contaminated with SF risks being classified as a high-risk medical device (Class III or equivalent), owing to the diverse biological properties and potential for SF-associated allergic reactions, necessitating extensive trials. Currently, there are no SS-only Phase II/III trials available. Researchers have identified fibroin-deficient mutant cocoons that can produce pure SS by complete dissolution at higher temperatures. This process results in hydrogels causing less than 1% inflammation. Achieving over 99% purity *via* enzymatic methods or lithium bromide treatment is crucial, but scaling production remains difficult, with yields dropping by 20% (Aramwit and Sangcakul, 2007). To tackle immunogenicity concerns, approaches include using mutant silkworms to generate transparent, elastic, and biocompatible pure SS hydrogels; applying surface coatings such as hydrophilic PEG/SS; and utilising analytical techniques such as ELISA to detect SF traces at levels below 0.1%. For clinical applications in wound dressings, especially for diabetic ulcers, standardisation is crucial. Recent GLP studies verify that SS with 99.5% purity is safe for these uses (Veiga et al., 2024).

### 7.2 *In vivo* biological assessment of sericin

**FIGURE 7:**
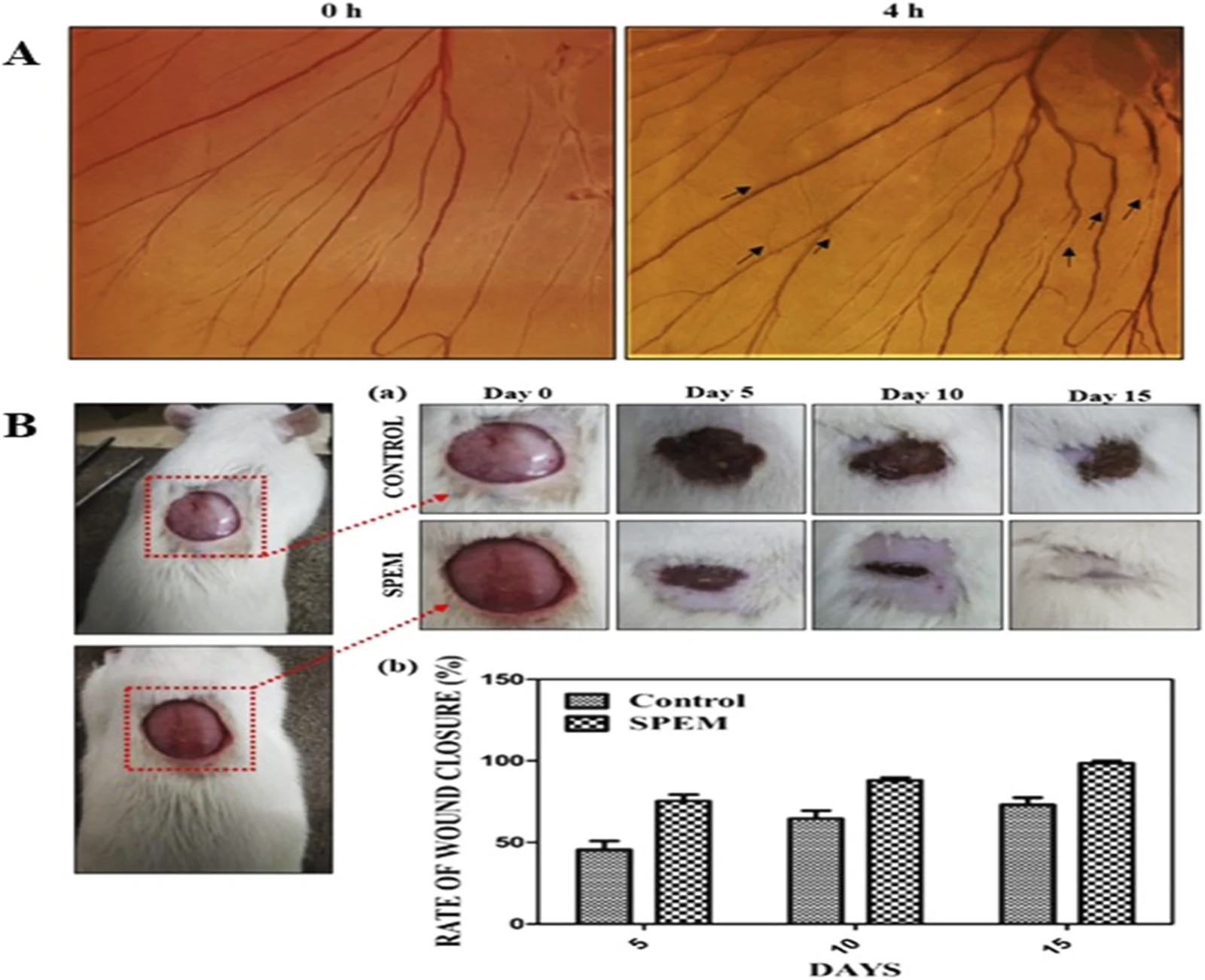
**(A)** Photographs of *in vivo* CAM assay in the presence of SPEM scaffold. Black arrows indicate the new blood vessels formed after 4 h of treatment. **(B)** (a) *In vivo* wound healing images of control and SPEM-treated groups on days 5, 10, and 15, respectively. (b) Percentage of wound closure in open excision rat wound model (Reproduced with permission from Bhoopathy et al. originally Figure 11A, and Figure 12B (Bhoopathy et al., 2023)).

A study by Qin et al. evaluated the toxicity of water-extracted sericin in Wistar rats. The study showed no maternal toxicity and assessed the potential acute and developmental toxicity of sericin when administered orally, finding no adverse effects on fetal development or teratogenicity at any doses. The study concluded that sericin extracted from *Bombyx mori* cocoons is safe at doses up to 1,000 mg/kg/day from day 7 to day 20 during pregnancy, supporting its potential for biomedical and nutraceutical applications (Qin et al., 2020). Mumtaz et al. (2023) study employed a D-galactose-induced aging mice model to assess the antioxidant and anti-aging properties of silk sericin. Sericin was given orally or in combination with D-galactose to eight groups of male Swiss albino mice. Although the animals showed symptoms of aging and oxidative stress, sericin significantly elevated antioxidant levels, strengthened the immune system, and reversed histological damage in the kidney, liver, and brain. Additionally, sericin therapy enhanced tissue structure and increased telomere length expression (Mohanty et al., 2025). Hong et al. (2021) conducted a study on the potential of silk sericin to regenerate bone in rats with type 1 diabetes. Three groups of rats were created: one received gelatin sponge with sericin, another received only gelatin, and the third received no graft. The group treated with sericin exhibited the greatest new bone volume and notable bone growth after 8 weeks. The study suggests that sericin greatly improves bone regeneration in diabetics, probably due to its capacity to increase angiogenesis and regulate immune responses (Hong et al., 2021). Kadhim and Rajab (2024) evaluated paclitaxel-loaded sericin nanoparticles as a novel pulmonary drug delivery system using self-assembled sericin nanoparticles (NPs) combined with poloxamer 407 to deliver to the lungs. *In vivo,* examination of the aerosolised nanoparticles in the lungs established significantly improved lung targeting, increased drug accumulation, and prolonged drug retention compared to intravenous Taxol (Kadhim and Rajab, 2024). A study on sericin found that it has antihyperglycemic effects, as it reduces blood glucose levels in diabetic rats, increases body weight, and provides protective effects on liver function and lipid profiles, indicating potential for diabetes management (Pachhiappan et al., 2023). Pandey et al. (2024) reported, in Wistar rats, that the effectiveness of resveratrol-loaded sericin hydrogel in wound healing. The group treated with sericin hydrogel loaded with resveratrol demonstrated reduced TNF-α levels, faster re-epithelialization, and accelerated wound contraction. This shows that sericin-resveratrol hydrogel enhances wound healing by stimulating tissue regeneration, reducing inflammation, and providing antimicrobial protection (Pandey et al., 2024). Lee et al. (2023) studied the comparison of collagen and gelatin scaffolds for delivering silk sericin in bone regeneration using a rat calvarial defect model. The study focused on optimizing the scaffold material to maximize the bioactive effects of the silk sericin. Twelve rats were divided into two groups: one received gelatin sponges, and the other received collagen membranes. After implanting grafts, the animals were monitored for 8 weeks. Results showed that collagen had significantly greater new bone volume and more complete defect closure, leading to higher bone regeneration. The study identified that the collagen-sericin combination provides a more effective microenvironment for repairing bone defects compared to gelatin-based combinations (Lee et al., 2023). Reports shows development of sericin-covalently modified valve (SCMV) through coupling maleylated silk sericin with thiolated decellularized heart valves (DHV) *via* a Michael addition reaction to create chemically modified an advanced tissue-engineered heart valve. The SCMV showed a smooth surface and higher hydrophilicity compared to the decellularized value Heart (DHV), which is crucial for reducing platelet adhesion and preventing blood clotting in tissue-engineered heart valves. It also significantly promoted the adhesion of endothelial cells (ECs) under both static and flow conditions, ensuring the heart valves can integrate well with the body and function effectively. Silk sericin stimulates endothelial cells (ECs), leading to increased RhoA activation, Rho-associated protein kinase 2 (ROCK2) expression, and phosphorylated myosin light chain 2 (MLC2), which contribute to actin polymerisation and enhanced cell adhesion. Several integrin subunits were identified involved in the regulatory processes influenced by silk sericin, highlighting its complex interactions with endothelial cells (ECs). The findings underline the potential of silk sericin in tissue engineering applications and its promise in promoting endothelialization of decellularized heart valves (Song et al., 2024). Sericin, a biomaterial with low immunogenicity, excellent biocompatibility, cell adhesion, and antioxidant properties, has been shown to enhance fibroblast proliferation, stimulate cell migration, induce collagen production, and exhibited to be used in wound dressings and biomedical applications in combination with other polymers. Bhoopathy et al. (2023) developed sericin/human placenta-derived extracellular matrix (SPEM) scaffolds for wound healing efficient, which were evaluated *in vivo* using HaCaT cells, a chick embryo model, and a Wistar rat model. They demonstrated that SPEM treatment significantly improved wound healing rates in open excision wounds, achieving a 98% closure rate after 48 h, as shown in a scratch wound assay using HaCaT cells (Bhoopathy et al., 2023). The findings reported in an earlier study are illustrated in Figure 7.

**FIGURE 8:**
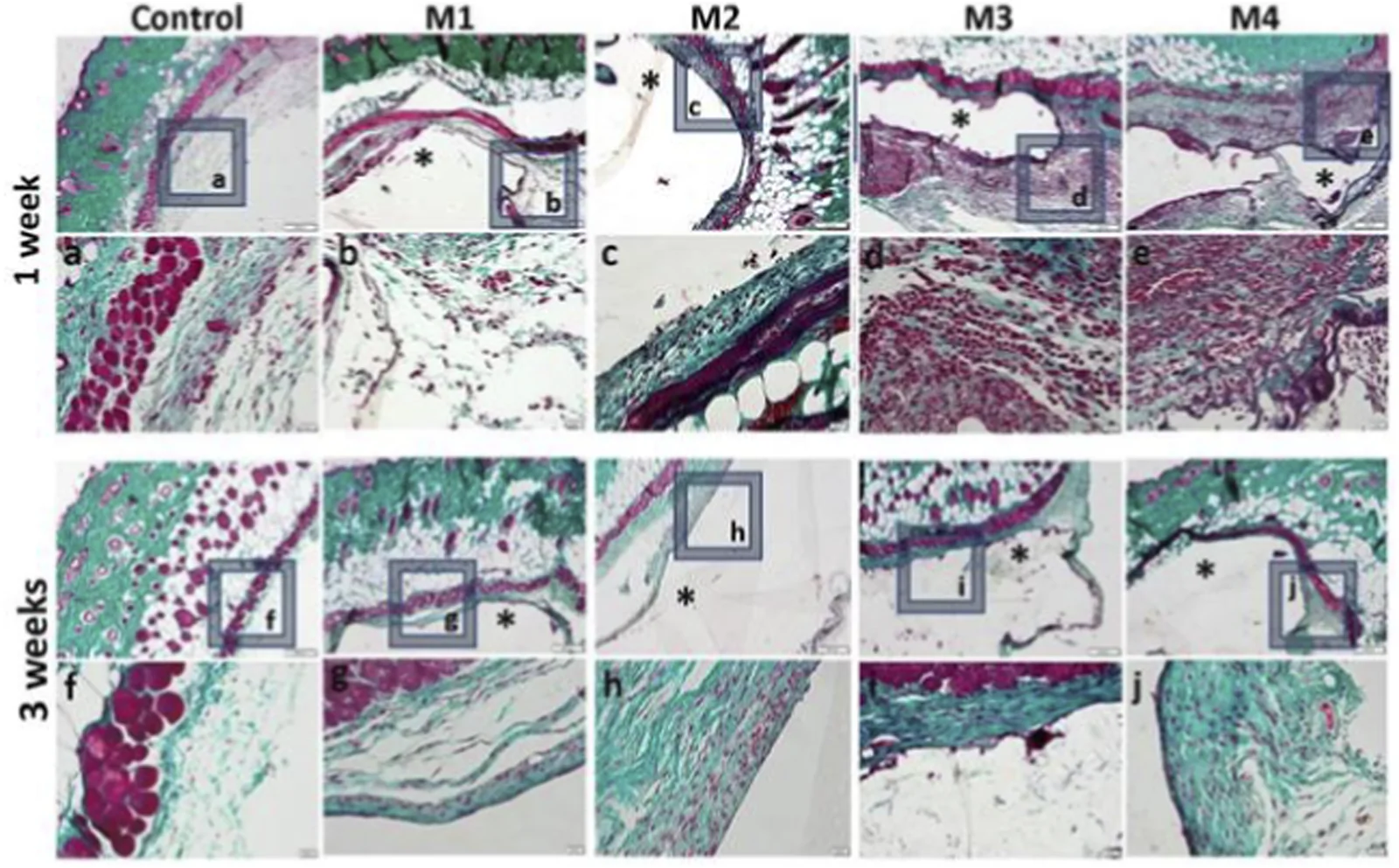
Histological analysis of the biocompatibility of HEMA/AMPSA hydrogels supplemented with fibroin/sericin, at 1 and 3 weeks after subcutaneous implantation (control group-without any material; M1 group-material containing MMT-5 mol/L-H95%-A5% with Sericin and Fibroin; M2 group-material containing MMT-5 mol/L-H97%-A3% with Sericin and Fibroin; M3 group-material containing MMT-5 mol/L-H95%-A5%; M4 group-material containing MMT-5 mol/L-H97%-A3%; * material; Gomori Trichrome staining, ob.4×, 20×; a-j 20× magnifications of ×4 pictures). (Reproduced with permission from Șerban et al. originally Figure 14 (Șerban et al., 2022)).

**FIGURE 9:**
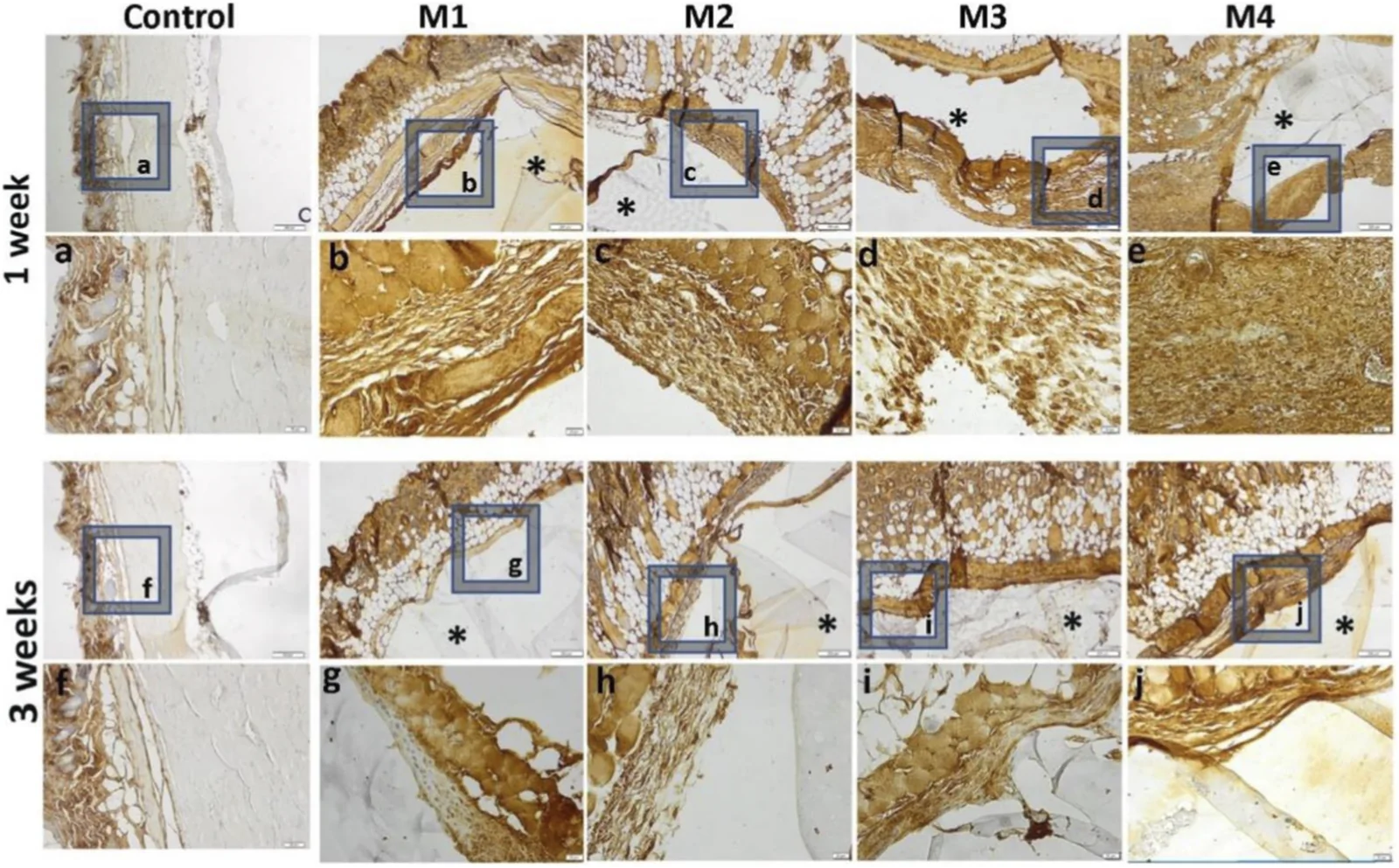
Immunohistochemical expression of TNFα and tissue-specific distribution at 1 week and 3 weeks post-implantation (control group-without any material; M1 group-material containing MMT-5 mol/L-H95%-A5% with Sericin and Fibroin; M2 group-material containing MMT-5 mol/L-H97%-A3% with Sericin and Fibroin; M3 group-material containing MMT-5 mol/L-H95%-A5%; M4 group-material containing MMT-5 mol/L-H97%-A3%; * material; ob.4×, 20×; a-j 20× magnifications of 4× pictures). (Reproduced with permission from Șerban et al. originally Figure 15 (Șerban et al., 2022).

Șerban et al. (2022) study evaluated the potential of newly developed nanocomposite hydrogels made from poly (2-hydroxyethyl methacrylate-co-2-acrylamido-2-methylpropane sulfonic acid) with modified clay, sericin, and fibroin for soft tissue engineering applications. The nanocomposite hydrogels, which were implanted into mice demonstrated biocompatibility and low cytotoxicity. Inflammatory reactions were more severe in materials without sericin and fibroin, but sericin hydrogels reduced inflammation and enhanced wound healing (Șerban et al., 2022). Șerban et al. reported an *in vivo* study evaluating the biocompatibility, inflammatory, and soft-tissue regenerative properties of the HEMA/AMPSA (poly (2-hydroxyethyl methacrylate-co-2-acrylamido-2-methylpropane sulfonic acid)) nanohydrogels after subcutaneous implantation in mice. The silk-based hydrogels (M1 and M2) containing sericin and fibroin were tested and compared with the single hydrogels (M3 and M4) that did not contain silk proteins. Test samples after dorsal subcutaneous implantation, observed at 1 and 3 weeks. Observation shown that all implanted materials elicited an initial inflammatory reaction, as was customary at the 1-week time point. However, the inflammatory responses of M1 and M2 (sericin- and fibroin-enriched hydrogels) were significantly lower than those of the others (M3 and M4). In conclusion, hydrogels that did not contain sericin or fibroin showed higher infiltration of polymorphonuclear cells and more intense acute inflammation, whereas the other hydrogels with higher protein content showed better tissue adaptation and a lower degree of acute inflammation. At 3 weeks, a moderate foreign body reaction was observed, with the presence of macrophages, lymphocytes, and occasional, prominent multinucleated giant cells, especially for surface of the material with coated with sericin and fibroin. Higher magnification of the implants revealed that the fibrous capsule and collagen deposition around the implants are significantly thinner in M1 and M2 than in M3 and M4 (Figure 8), confirmed from the results of trichrome staining. The less fibrous response observed with the sericin- and fibroin-containing hydrogels indicates that the products are more biocompatible, since the presence of large amounts of collagen and a thick fibrous capsule indicates poor integration of the implant and a chronic foreign body response. In addition, expression of the inflammatory cytokine TNF-α was assessed using immunohistochemistry in the vicinity of the implants to estimate the activity of inflammatory cytokines surrounding the implants. Moreover, M3 and M4 exhibited significant TNF-α staining, suggesting higher inflammation than M1 and M2, which exhibited lower TNF-α staining, suggesting less inflammation and increased tissue tolerance (Figure 9) (Șerban et al., 2022).

## 8 Conclusion

Sericin is a hydrophilic, naturally derived protein from silkworm cocoons, obtained as a byproduct of silk processing industries. It surrounds and bonds fibroin protein silk fibers within the silkworm cocoon. Several methods of degumming, including heat, alkali, acid, and enzyme treatments, can be used to remove sericin from silk fibers; however, the common industrial process involving heat and alkali often results in weakened biophysical properties of the sericin protein. Sericin exhibits non-toxic, biocompatible, and biodegradable qualities. Because of its unique set of beneficial physicochemical and biological properties, sericin is considered a highly promising material for various biomedical applications. Sericin is one of the newest potential biomaterials in tissue engineering, as it offers a unique set of beneficial biological properties that make it suitable for various pharmacological, biomedical, and biotechnological applications. Sericin exhibits anti-tyrosinase, anticoagulant, and anti-cancer activities. It resists oxidation, is antibacterial, anti-apoptotic, and UV resistant, which makes it useful in skincare (cosmetics). Sericin promotes cell growth and proliferation in serum-free media. Different forms of sericin biomaterials, including powder, paste, film/membrane, porous scaffold, solution, beads, fibers, and nanoparticles, are used across diverse biomedical fields. Recently, interest has increased in chemically modifying sericin to improve its mechanical strength and stability. In the future, more advanced *in vitro* and animal clinical studies are needed to explore and confirm sericin’s capabilities, supporting its potential use. This review introduces sericin in various forms for different biomedical applications, highlighting its potential as a versatile material. Its broad properties and processed forms suggest a promising future for this bio-based polymer, especially with emerging products like wound dressings. However, challenges remain, such as improving its mechanical properties to better serve as a synthetic substitute, developing effective drug delivery systems, and ensuring sterility for various implant applications (Arango et al., 2023; Jao et al., 2016). Sericin genes in *Bombyx mori* show distinct isoform-level features essential for biomedical uses. The main gene, Ser1, plays a key role in cocoon structure and spinning, with high-molecular-weight, hydrophilic, bioactive traits that boost cell adhesion, antioxidant activity, and scaffold formation. Ser2 produces more soluble and adhesive sericin fractions associated with early extraction, supporting biocompatibility and antioxidant functions. Ser3 increases sericin diversity and is crucial for silk gland secretion and developing biomaterials with good film- and hydrogel-forming abilities. Ser4 and Ser5 are recent genes/proteins that broaden understanding of silk sericin and may represent significant fractions that were previously discovered, although their functions in biomedicine are still being studied. The newly identified Ser6 (P150/SP150), expressed in the middle silk gland and present in pre-cocoon silk, is highly hydrophilic and could become a new source for functional silk biomaterials, with research biomedical applications still under investigation. Overall, these genes demonstrate that sericin is not a single protein but a family of isoforms, Ser1–Ser6, with varying molecular weights, solubilities, and biological functions. This diversity affects the development of wound dressings, hydrogels, films, scaffolds, and drug-delivery systems. Furthermore, this genetic variation explains the different performances of sericin materials extracted through various methods in tissue engineering and antibacterial uses.
